# SpinDynamica: Symbolic and numerical magnetic resonance in a Mathematica environment

**DOI:** 10.1002/mrc.4642

**Published:** 2017-09-20

**Authors:** Christian Bengs, Malcolm H. Levitt

**Affiliations:** ^1^ School of Chemistry University of Southampton Southampton SO17 1BJ UK

**Keywords:** Mathematica, NMR, numerical calculation, symbolic programming

## Abstract

SpinDynamica is a set of Mathematica packages for performing numerical and symbolic analysis of a wide range of magnetic resonance experiments and phenomena. An overview of the SpinDynamica architecture and functionality is given, with some simple representative examples.

## 1 INTRODUCTION

*Mathematica* is a symbolic computational system, which was launched in 1998 and has grown into a highly successful and widely used computational platform in numerous scientific fields, including mathematics, engineering, physics, design, statistics, and geography.(1) It is a very general “high‐level” system that supports a wide range of user expertise and provides seamless access to expert numerical and symbolic algorithms, graphical and animation tools, and specialized software packages. It is also platform‐independent to a high degree.

*SpinDynamica* is a computational system for (mainly nuclear) spin dynamics within Mathematica, programmed mainly by Malcolm H. Levitt, with relatively minor contributions by Andreas Brinkmann, Jyrki Rantaharju, and Soumya Singha Roy, and recent substantial coding by Christian Bengs. SpinDynamica takes immediate advantage of the Mathematica environment (programmability, generality, platform‐independence, access to a wide range of mathematical, statistical and graphical tools, etc.). SpinDynamica provides a palette of “toolbox”‐like routines, which may be deployed by the magnetic resonance research community, as well as a small number of powerful “top‐level” routines, which allow common classes of spin dynamical computations to be performed with minimal user preparation. SpinDynamica grew out of some early Mathematica routines programmed by Levitt to support the theoretical elements of his textbook *Spin Dynamics: Basics of Nuclear Magnetic Resonance*.(2) Since then, it has grown in ambition, generality, and power—although at a slow pace, limited by the time available for what is essentially a side project.

The central aim of SpinDynamica may be summarized as follows: “If you can write down the Hamiltonian (or the relaxation superoperator) of a spin system, in the form of an equation, then you can simulate the experiment.” The user community may judge how well this central aim has been satisfied, so far.

The use of Mathematica has advantages and disadvantages. The main advantages are the platform‐independence, seamless access to an enormous range of other functions and packages, a relatively high level of future‐proofing against upgrades in the system and Mathematica software and other features of the computational environment, without direct intervention from the SpinDynamica programmers themselves. Since the programmers are not experts in numerical mathematics or in system software and have insufficient resources for extensive software maintenance, this is a very important advantage. Furthermore, the pattern‐based and modular “philosophy” of Mathematica, as advanced by Wolfram's ambitious book *A New Kind of Science*,(3) does suit the field of magnetic resonance rather well.

The choice of Mathematica as a platform does come with disadvantages. (a) Mathematica is commercial software with a hefty license fee for single users. This is an unavoidable problem that is only partially alleviated by the patchy availability of university site licenses. The SpinDynamica code itself, on the other hand, is free and open‐source. (b) Mathematica does not have a reputation for numerical efficiency: benchmark speed comparisons of SpinDynamica with other computational packages such as *SIMPSON*,(4) *SPINEVOLUTION*,(5) or *SPINACH*(6) are likely to prove embarrassing for proponents of SpinDynamica. The relatively slow execution speed and high memory demands of SpinDynamica are not necessarily intrinsic features of the Mathematica platform: These weaknesses may mainly be attributed to a lack of expertise on the programmer's part in generating code, which is both general and also fast in execution – itself a result of time limitations for the SpinDynamica programmers, given that this has always been a side project that has taken place on the side of other duties. (c) Although most advanced users find Mathematica to be an impressive and elegant computational environment (once the steep learning phase is overcome), Mathematica code does have the tendency to become quite unreadable to a reader—even to the programmer him/herself after some time away. The SpinDynamica source code tries to alleviate this problem by using informative symbol names (often rather long) and by including commentary. Nevertheless, the opaqueness of some SpinDynamica source code cannot be denied. Fortunately, most users will never have to delve into the inner workings of the SpinDynamica routines.

SpinDynamica should not be viewed as a competitor to well‐optimized numerical codes such as *GAMMA*,(7) *SIMPSON*,(4) *SPINEVOLUTION*,(5) *BlochLib*,(8) *EASYSPIN*,(9) or *SPINACH*,(6) most of which have been designed for specific purposes (solid‐state NMR computations, in the case of *SIMPSON* and *SPINEVOLUTION*, EPR spectra, in the case of *EASYSPIN*). If the user requires rapid simulations of solid‐state NMR experiments, then SpinDynamica is not the tool of choice. The strength of SpinDynamica is in its capability of performing analytical and symbolic calculations, and as a valuable assistant in helping understand and visualize the spin‐dynamical concepts underlying NMR experiments and to develop and test new experimental concepts on that basis. It is regularly used for this purpose in our research group. Some literature examples of SpinDynamica calculations may be found in references herein.(10, 11, 12, 13, 14, 15, 16, 17, 18, 19, 20)

The authors are aware of several other projects for the treatment of nuclear spin dynamics in Mathematica.(21, 22, 23, 24, 25) While these pieces of software are undoubtedly very useful for specific classes of spin dynamical problems, SpinDynamica has a broader remit.

SpinDynamica is not a complete project and is under active development. The current paper (which is the first on the topic of SpinDynamica) refers to version 3.0.1, released in 2017 through the website http://www.spindynamica.soton.ac.uk. The interested reader should download the latest version of SpinDynamica and work through the included documentation. The examples given in the current paper may also be downloaded from the SpinDynamica website.

SpinDynamica contains a large number of symbols. This article only discusses a representative selection. Furthermore, most symbols used by SpinDynamica have multiple functionality (through the so‐called function overloading). This article only discusses the representative basic functionality for each symbol and is far short of being a complete manual for SpinDynamica. More complete information on a given symbol is obtained by executing ?<symbol> within a SpinDynamica session.

## 2 ARCHITECTURE OF SPINDYNAMICA

Although most users of SpinDynamica should not have to trouble themselves with the internal anatomy of the program, a brief overview of the program architecture may be of interest and is sketched here.

**Figure 1:**
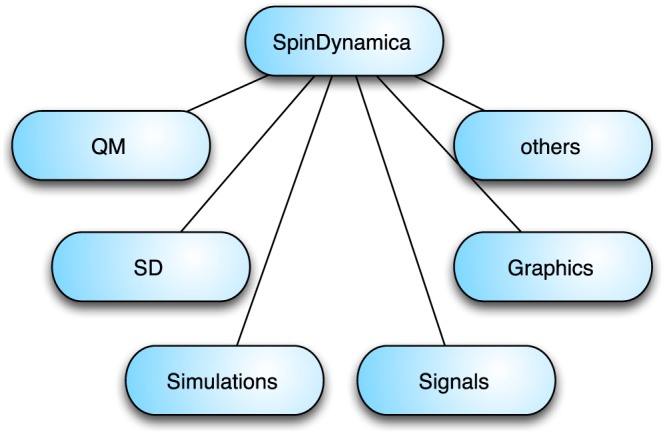
Overall organization of SpinDynamica. The quantum mechanics packages are denoted “QM” and the spin dynamics packages are denoted “SD”

SpinDynamica is organized as hierarchical structure of Mathematica packages, which interact with each other and call each other as needed. The overall structure is shown in Figure 1. The top‐level SpinDynamica package may access a set of lower‐level packages, labeled “QM” (for quantum mechanics), “SD” (for spin dynamics), as well as specialized packages (and/or sets of packages) associated with high‐level simulation functions, the processing and plotting of signals, various useful graphical objects, and a miscellany of other functions, including routines for handling matrices of various types, implementing rotations, axis systems, Euler angles, Wigner matrices, Clebsch‐Gordan coefficients, and various tensor manipulations.

**Figure 2:**
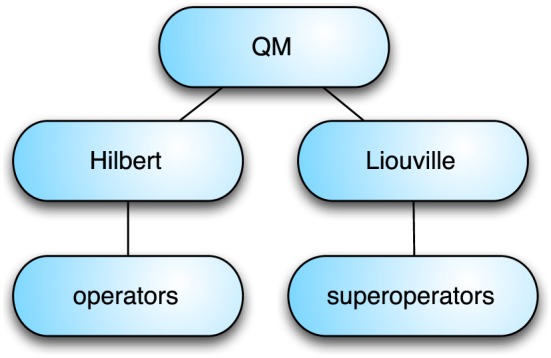
Organization of the quantum mechanics (QM) packages of SpinDynamica

The structure of the QM packages is shown in Figure 2. The Hilbert package provides the definitions needed to set up spin systems and sets of basis states, and interacts with an additional package containing the definitions and functionality of numerous types of nuclear spin operator. The Liouville package implements general operator bases and implements the functionality of several important superoperators used in magnetic resonance theory.

The QM packages are often found to be particularly helpful for those magnetic resonance researchers who are interested in the detailed theory of nuclear spin systems and its mathematical formalism. Some simple examples are given below.

**Figure 3:**
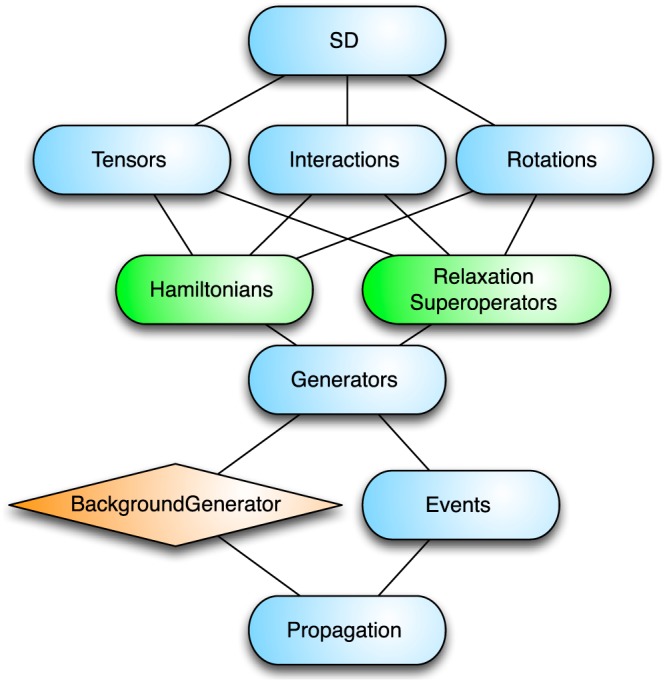
Organization of the spin dynamics (SD) packages of SpinDynamica

The structure of the SD packages is shown in Figure 3. This set of packages includes routines for constructing nuclear spin Hamiltonians and relaxation superoperators using tensor mathematics, rotations, Euler angles, Wigner matrices, and other elements of angular momentum theory. SpinDynamica includes tables of useful nuclide properties such as nuclear spin quantum numbers and magnetogyric ratios. Functions for calculating the dipole‐dipole coupling tensors from static molecular geometry are also provided.

In SpinDynamica, the term generator refers to a spin Hamiltonian or a relaxation superoperator. A generator may be time‐dependent; functions are provided for expressing the time‐dependence of a generator in terms of global or local time variables, as described below. Periodic time‐dependence may also be indicated and triggers special treatment in numerical computations. SpinDynamica allows the combination of several different generators, taking into account their time‐dependence or periodicity.

An NMR experiment often involves the execution of a set of events, which are chained together in chronological sequence. Each event may consist of a spin Hamiltonian, or a relaxation superoperator, or both, acting for a certain amount of time. As described below, an event may also comprise an instantaneous manipulation of the nuclear spin system, such as rotation of spin states by an infinitely short pulse, or more abstract constructions, such as the filtration of the spin density operator according to a set of coherence orders. The Events package of SpinDynamica contains functionality for chaining and combining sequences of events, respecting the time‐dependence and/or periodicity of the corresponding generators.

The Propagation package of SpinDynamica contains the routines for numerical propagation of the spin density operator under arbitrary event sequences and generators. Numerical propagation is usually executed by passing the information to the major Mathematica routine NDSolve, which provides the numerical solution of differential equations. This approach allows the solution of time‐dependent propagation without user‐defined “time‐slicing” and provides access to the numerous algorithms and numerical mathematical techniques incorporated into the powerful NDSolve routine. This approach also ensures that future advances in numerical mathematics achieved by the Mathematica programmers are automatically made available to SpinDynamica.

Figure 3 indicates the importance of the SpinDynamica symbol BackgroundGenerator. This symbol is described in Section 8.2 and is used to specify a Hamiltonian, relaxation superoperator, or combination of both, that acts continuously “in the background" throughout the spin propagation. As shown in the examples below, the BackgroundGenerator is often used to represent the “internal” Hamiltonian, or relaxation superoperator, of the spin system, while the event sequence represents the interactions with fields applied by the apparatus.

**Figure 4:**
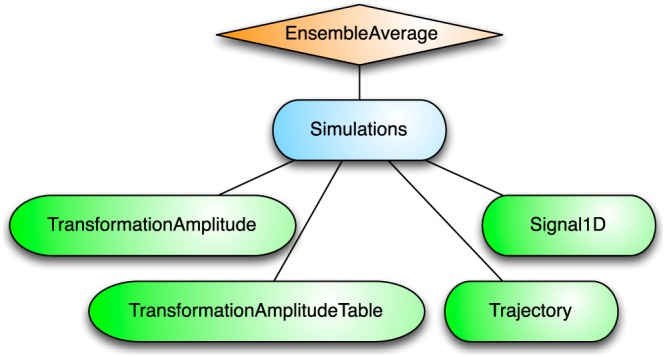
Organization of the simulation packages of SpinDynamica

As shown in Figure 4, the Simulation package of SpinDynamica contains a small number of high‐level routines, which allow common NMR simulation tasks to be conducted with minimal user programming. In many cases single‐line instructions are sufficient to perform useful calculations. At the time of writing, these top‐level simulation routines include (a) TransformationAmplitude, which is typically used to determine the amplitude of conversion of one spin operator into another by a given sequence of events, (b) TransformationAmplitudeTable, which allows exploration of the dependence of a transformation amplitude on one or more parameters, (c) Trajectory, which allows the coefficients of one or more spin observables to be tracked continuously through a sequence of events, and (d) Signal1D, which generates one‐dimensional NMR spectral information under a wide variety of circumstances. Routines are also provided for processing and plotting such data.

The important symbol EnsembleAverage is associated with each of these routines. This allows the calculations to be repeated for a set of values of one or more parameters, and the results combined (with arbitrary weights). By default, the multiple calculations deployed by EnsembleAverage are conducted in parallel by distributing the calculations over the multiple Mathematica kernels accessible to the local implementation. The parallel computation option may, of course, be suppressed if desired.

In the context of solid‐state NMR, a common application of EnsembleAverage is in powder averaging,(26) and SpinDynamica provides a variety of Euler angle sampling schemes for this purpose. However, EnsembleAverage allows a much broader range of applications, permitting the averaging over arbitrary distributions of spin interaction parameters or magnetic fields, for example. Mathematica contains powerful functionality for the sampling of multivariate distributions; these may be deployed to generate sophisticated multivariate sampling sets for the EnsembleAverage function.

As shown in Figure 1, SpinDynamica also contains additional packages for graphical objects, signal processing, specialized matrix‐vector routines, and three‐dimensional geometry.

## 3 GETTING STARTED

### 3.1 Using Mathematica

SpinDynamica requires a working and licensed version of Mathematica. The latest SpinDynamica release at the time of writing (3.0.1) is compatible with Mathematica version 11.1.1. It is likely to be compatible with earlier Mathematica versions, but this cannot be guaranteed. In the past, major new releases of Mathematica have required minor changes to SpinDynamica, in order to retain compatibility.

Before using SpinDynamica, users who are unfamiliar with Mathematica are strongly advised to acquire basic Mathematica skills, for example, by taking some of the introductory tutorials and video courses available at www.wolfram.com. Some basic Mathematica operations and techniques are sketched in the Appendix.

### 3.2 Using SpinDynamica

Running SpinDynamica requires the following steps:

1. The latest SpinDynamica release may be downloaded from www.spindynamica.soton.ac.uk. A complete release consists of a set of folders, containing documentation and examples, and a subfolder named SpinDynamica, which contains the code. The SpinDynamica folder and its contents may be placed anywhere on the host computer but must be kept intact and not modified in any way.
2. The $Path variable of Mathematica must be set in order to inform the kernel of the SpinDynamica location. This is typically done by executing an instruction of the form: AppendTo[$Path,<*SpinDynamica* location>].
3. SpinDynamica is loaded by executing a cell containing the instruction Needs[“SpinDynamica‘”]. A successful load returns an output similar to that shown below:

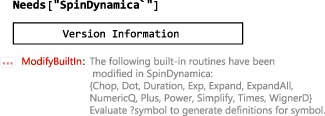

  The version number of a loaded SpinDynamica package may be examined at any time by executing $SpinDynamicaVersion.

Detailed installation instructions and examples are included in each release on www.spindynamica.soton.ac.uk.

An executable Mathematica notebook containing the examples in this paper may be downloaded as Supporting Information and will also be released on the SpinDynamica website.

## 4 SPIN SYSTEMS, BASES, AND OPERATORS

### 4.1 **SpinSystem**

SpinDynamica treats the dynamics of small clusters of spins, each of which may have any valid spin quantum number. Current versions of SpinDynamica (3.0.1 and earlier) can deal comfortably with up to five coupled spins‐1/2.

The first instruction in any SpinDynamica notebook, after loading the SpinDynamica package, is often to set the current spin system by executing an instruction of the type

SetSpinSystem[<spin system>]

where the <spin system> specification has the general form {{<lab#1,qunum#1>},{<lab#2,qunum#2>}...}. Each spin in the system has a *label* (lab), which may be an integer or a text string, and a *quantum number* (qunum), which may be an integer or a half‐integer. For example, a two‐spin system, consisting of one‐spin‐1/2 nucleus called “I,” and one spin‐1 nucleus called “S,” is set up as follows:

SetSpinSystem[{{“I”,1/2},{“S”,1}}]

Shortcuts are available for common tasks. For example, a system of three spins‐1/2, with labels {1,2,3} is constructed using the simple command:

SetSpinSystem[3]

The execution of a SetSpinSystem command defines the spin system for the rest of the Mathematica session, unless a new SetSpinSystem command is executed. The current spin system may be examined at any time by executing the command

SpinSystem[]

(the empty square brackets are necessary).

### 4.2 **Bases**

SpinDynamica performs many spin dynamical computations by using a Hilbert space spanned by a set of orthonormal spin states. Several preprogrammed bases are available. Bases may also be defined by the user.

The current Hilbert basis may be examined at any time by executing the command

Basis[]

(the empty square brackets are necessary).

#### 4.2.1 BasisDimension

The Hilbert basis dimension (number of states in the basis) is given by

(1) $$N_{H}=\overset{N}{\underset{j=1}{\prod }}(2I_{j}+1)$$

where the spin system consists of *N* spins with angular momentum quantum numbers {*I*_1_,*I*_2_…*I*_*N*_}. The dimension of the current basis is accessed by executing the command:

BasisDimension[]

#### 4.2.2 **ZeemanBasis**

Note: The examples in this section assumes prior execution of the command SetSpinSystem[2].

The default Hilbert space basis is the ZeemanBasis. Execution of the line below generates the indicated output:


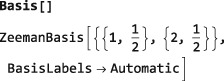


This indicates that the current Hilbert basis is a ZeemanBasis for a system of two spins‐1/2 with labels 1 and 2. The BasisLabels instruction is a more advanced feature, which is not discussed here.

The basis kets for the current basis may be examined by executing the command:

BasisKets[]

The kets are presented in conventional Dirac notation, using *α* and *β* symbols for spin‐1/2 states with angular momentum ±ℏ/2 along the z‐axis, respectively. For the two‐spin‐1/2 system, using the default Zeeman basis, the basis kets are as follows:


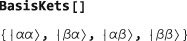


The third ket in the current basis may be extracted with the simple command:


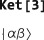


The basis bras are as follows:


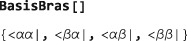


The products of the first bra with the second ket, and the third bra with the third ket, are computed as follows:


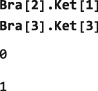


which illustrates the orthonormality of the basis.

#### 4.2.3 **SingletTripletBasis**

In the case of two spins‐1/2, a basis consisting of the spin‐0 singlet state and the three spin‐1 triplet states is useful, especially in the context of long‐lived nuclear spin states.(27) This basis may be set by executing

SetBasis[SingletTripletBasis[]]

and is comprised of the following set of basis kets:


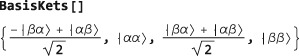


#### 4.2.4 Other basis routines

SpinDynamica provides many other routines for defining, manipulating, and constructing kets, bras, and Hilbert space bases. The available symbols include ProductKet, ProductBasis, Eigenbasis, DefineBasis, and BasisDimension. Refer to the SpinDynamica documentation for details.

### 4.3 Operators

Note: The examples in this section assumes prior execution of the command SetSpinSystem[3].

SpinDynamica supports a range of useful spin operators. Only a selection is documented here. Note that all operators are independent of the basis in which they are represented or constructed.

#### 4.3.1 **opI**

The main routine for defining nuclear spin operators in SpinDynamica is called opI(regrettably, the preferable symbol I is reserved in Mathematica for the imaginary number $\sqrt{-1}$). For example, the operator for the angular momentum along the z‐axis, for the spin with label 1, is denoted:

opI[1,“z”]

The quotation marks are necessary here, to indicate that the argument of opI is a text string “z”, and not a symbol with the name z. When opI is evaluated, the output format resembles ordinary scientific notation:


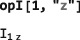


If the first argument (the spin label) is missing, SpinDynamica assumes summation over all spins in the current SpinSystem:


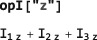


The second argument may be a string of the form “x”, “y”, or “z” (indicating the positive Cartesian axes), a string of the form “‐x”, “‐y”, or “‐z” (indicating the negative Cartesian axes), a string of the form “+” or “‐” (indicating the shift operators), or a string of the form “*α*” or “*β*”(indicating spin‐1/2 polarization operators(28)). The second argument may also be a single number or symbol, which is then interpreted as a phase angle in the xy‐plane, for example:


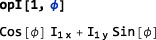


In this example, the Greek letter *ϕ* may be inputted in most Mathematica implementations through the keystrokes <esc>f<esc>.

Similarly, an angular momentum operator in a direction set by the polar angles {*θ*,*ϕ*} is generated using the following syntax:


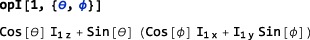


If any of these symbols have explicit values, the trigonometric functions are evaluated, for example:


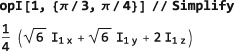


This example uses the Mathematica function Simplify to reduce the output to its simplest form.

The product of two or more angular momentum operators may be constructed using “.”, for example:

opI[1,“x”].opI[2,“y”]

SpinDynamica knows when two operators commute and reorganizes the operator product accordingly, as shown by the following example:


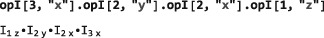


The scalar product of two angular momentum operators, such as **I**_1_·**I**_2_, is constructed by using “.” on two opI objects lacking the second argument:


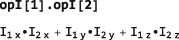


An operator opI, or indeed any operator in SpinDynamica, may be applied to a Ket object, as illustrated below:


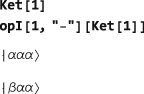


#### 4.3.2 Complex exponentials of operators

Spin dynamical calculations often involve the complex exponentials of operators, since these appear in the solutions for the spin propagation under the time‐dependent Schrödinger equation, in many important cases. SpinDynamica is comfortable with complex operator exponentials. The example below shows the complex exponential of a product operator in a two‐spin‐1/2 system, applied to a particular Ket:


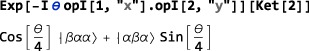


Note the symbolic solution in this case.

#### 4.3.3 **RotationOperator**

Rotation operators are given by complex exponentials of angular momentum operators, for example,

(2) $$R_{jx}(\beta )=\exp\{-i\beta I_{jx}\}$$

which represents the rotation of the spin angular momentum of spin *I*_*j*_ by an angle *β* about the x‐axis. Rotation operators are often used to represent the idealized actions of radiofrequency pulses and are implemented in SpinDynamica by the symbol RotationOperator (the synonym opR may also be used).

The syntax for a RotationOperator about a single Cartesian axis is given by:

$$\begin{array}{ccc} & \text{RotationOperator[} & \\ & \text{<spins>,\{<angle>,<axis>\}}\\ & \text{]} \end{array}$$

Omitting the first argument defines a RotationOperator acting on all spin operators, while specifying a single spin label or a list of spin labels defines a RotationOperator for one or more spins. The <angle> argument may either be numeric or symbolic. Valid <axis> specifications are the same as the axis specifications for the angular momentum operators, opI.

Consider, for example, a three‐spin system with two protons and one carbon‐13. It is convenient to define a symbol Ispins for the protons and a symbol Sspins for the ^13^C nuclei. Selective rotation operators for ^1^H or ^13^C nuclei may then be implemented as follows:


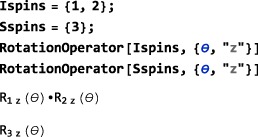


The operator for a rotation by an <angle> about an arbitrary axis in the xy plane is defined by using a <phase> angle, as follows:

$$\begin{array}{ccc} & \text{RotationOperator[} & \\ & \text{<spins>,\{<angle>,<phase>\}}\\ & \text{]} \end{array}$$

for example:


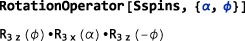


SpinDynamica expresses a rotation about an axis in the xy‐plane as a “sandwich” of three rotations about Cartesian axes.(2)

In a similar manner, one can define a RotationOperator for a rotation about an arbitrary axis in three dimensions. The orientation of the axis is defined by polar angles (*θ*,*ϕ*). The angle *ϕ* (<phase>) indicates the orientation in the xy plane and *θ*(<tilt>) indicates the deviation from the z‐axis:

$$\begin{array}{ccc} & \text{RotationOperator[} & \\ & \text{<spins>,\{<angle>,\{<phase>,<tilt>\}\}}\\ & \text{]} \end{array}$$

A RotationOperator may be applied directly to a Ket, for example:


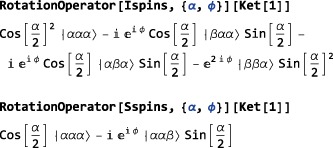


Notice how SpinDynamica applied the first rotation only to spin‐states of spins 1 and 2, while the second rotation has been applied to spin‐states of the third spin.

#### 4.3.4 **MatrixRepresentation**

Note: The examples in this section assume prior execution of the command SetSpinSystem[2].

The matrix representation of an operator in a given Hilbert basis may be computed as follows:

MatrixRepresentation[<operator>,<basis>]

where the current basis (defined by SetBasis, and revealed by executing Basis[]) is used if the <basis> argument is missing. The matrix representation of an operator is a *N*_*H*_×*N*_*H*_ square matrix. For example, the matrix representation of the scalar product operator **I**_1_·**I**_2_, in the singlet‐triplet basis for a pair of spins‐1/2, is computed as follows:


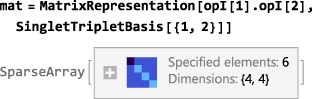


The “=” syntax computes the right‐hand side of the equation and assigns the result to the left‐hand side, in this case, the symbol mat (which therefore changes from blue to black when the assignment has been made). The output of MatrixRepresentation is a matrix stored in SparseArray format, which is memory‐efficient for large matrices. The format may be converted to a readable format by applying the Mathematica symbol MatrixForm:


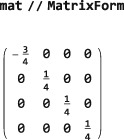


A graphical representation of the matrix may be generated using the Mathematica symbol MatrixPlot:


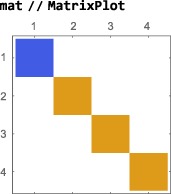


This format is useful for visualizing the structure of large, complex, matrices. As an example, here is a graphical matrix representation of the operator $\exp\{-i(\pi /4)4I_{1x}I_{2y}I_{3x}\}$ in the Zeeman basis of a five‐spin‐1/2 system: 
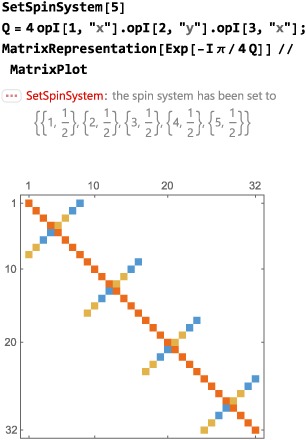


#### 4.3.5 **Operator**

Note: The code in this section assumes prior execution of the command SetSpinSystem[2].

An arbitrary spin operator may be defined in SpinDynamica by specifying its matrix representation in a given basis, using the Operator symbol. The syntax is as follows:

Operator[<matrix>,<basis>]

Here, <matrix> specifies a square matrix of the following form: {{a,b,…},{c,d,…}...}, while <basis> indicates the basis in which the matrix representation of the operator is equal to <matrix>. If <basis> is omitted, the current Hilbert basis is assumed.

The example below uses the Mathematica routine RandomInteger to generate a matrix with random entries and then defines an operator, which has this matrix representation in the ZeemanBasis:


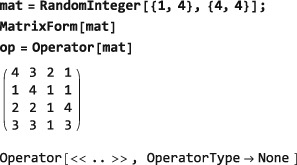


An operator generated by applying Operator to a matrix in a given basis is itself basis‐independent. The matrix representation of an Operator object may be generated in any basis by using MatrixRepresentation, just like any other operator. The MatrixRepresentation of the same Operator takes the following form in the SingletTripletBasis:


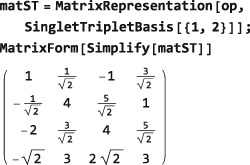


#### 4.3.6 More operators

SpinDynamica contains many other spin operator definitions, including the following:

- UnityOperator
- NullOperator
- SingleTransitionOperator
- SphericalTensorOperator
- ProjectionOperator
- ShiftOperator
- SpinPermutationOperator

These are described in the documentation accompanying a SpinDynamica release. Usage messages are generated by executing ?SingleTransitionOperator, etc.

### 4.4 Manipulations of operators

The code in this section assumes prior execution of the command SetSpinSystem[5].

#### 4.4.1 **OperatorAmplitude**

Consider the following common problem: For two spin operators *A* and *B*, express *A* in the following form:

(3) A=b×B+(operators orthogonal toB)

where *b* is a number, and the term “orthogonal,” as applied to operators, means that their matrix representations have the property Tr{*A* ^*†*^ *B*}=0.

The number *b* in the above equation has been termed the *operator amplitude* of *B* in *A*, and denoted:(20)

(4) b=(A→B)

Loosely speaking, the operator amplitude (*A*→*B*) indicates “how much of the operator *B* is present in the operator *A*.”

The SpinDynamica routine OperatorAmplitude determines the operator amplitude of any one operator in another. For example, since the operator *I*_*y*_ may be expressed as

(5) $$I_{y}=\frac{i}{2}\left(I^{-}-I^{+}\right)$$

then the operator amplitude of *I* ^+^ in *I*_*y*_ is given by (*I*_*y*_→*I* ^+^)=−*i*/2, as follows:


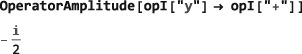


The computation is trivial in this simple case but less so in more complex situations. The following example shows how the amplitude of the (−3)‐quantum operator $I_{1}^{-}I_{2}^{-}I_{3}^{-}I_{4}^{+}I_{5}^{-}$ is extracted from a complex product operator expression:


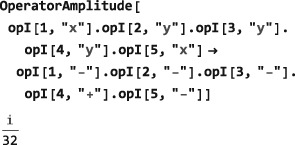


In the example below, a thermal equilibrium density operator is calculated for a set of protons in a magnetic field of 11.4 Tesla and temperature of 300 Kelvin, and the symbol Meq set equal to the coefficient of the angular momentum operator in the field direction:


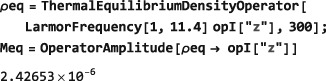


The ThermalEquilibriumDensityOperator symbol is explained in Section 7.1. As is well known, the thermal equilibrium nuclear magnetization is a very small quantity, in ordinary circumstances.

#### 4.4.2 **CoherenceOrder**

The code in this section assumes prior execution of the command SetSpinSystem[3].

The SpinDynamica routine CoherenceOrder analyzes an operator to determine the orders of coherence, which would be present, if that operator was a spin density operator representing the state of the spin ensemble. The syntax has the form

CoherenceOrder[<spins>,<operator>]

where the <spins> argument is a single spin label, or list of spin labels, and <operator> is an operator. If <spins> is missing, it is assumed that all spins in the current spin system are included. For example, the operator $I_{1}^{+}I_{2}^{+}I_{3}^{-}$ is classed as a (+1)‐quantum operator if all spins are considered:


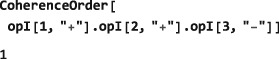


However, it is a (+2)‐quantum operator if only the first two spins are considered:


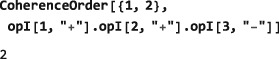


If an operator contains several coherence orders, a list of those coherence orders is returned:


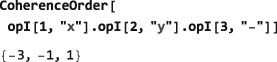


#### 4.4.3 **OperatorNorm** and **NormalizeOperator**

The code in this section assumes prior execution of the command SetSpinSystem[1].

The norm of an operator is given by the square root of the sum of the square magnitudes of its matrix elements:

(6) $$\left\|Q\right\|={\left\{{\underset{r,s}{\Sigma |Q_{rs}|}}^{2}\right\}}^{1/2}$$

The norm of an operator depends on the dimension of Hilbert space and, hence, the current value of SpinSystem[].

In SpinDynamica, the norm of an operator may be calculated using the symbol OperatorNorm:


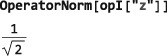


Any operator may be normalized by dividing it by its norm. The symbol NormalizeOperator performs this task:


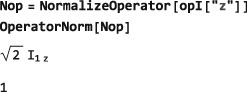


## 5 **SUPEROPERATORS**

The code in this section assumes prior execution of the command SetSpinSystem[2] and SetOperatorBasis[CartesianProductOperatorBasis[]].

Superoperators are objects that act on operators. They may either return the same operator or a new operator. Superoperators are essential for the description of advanced NMR experiments, especially those involving motional processes, and are very convenient for describing some common NMR methods, such as coherence order filtration by phase cycling or field gradient pulses.(28, 29) In this paper, superoperators are indicated by a hat.

### 5.1 **CommutationSuperoperator**

The commutation superoperator of an operator *Q*_1_ is denoted ${\hat{Q}}_{1}$. Application of the superoperator ${\hat{Q}}_{1}$ to a second operator *Q*_2_ generates the commutator of the two operators.(29)

(7) $${\hat{Q}}_{1}Q_{2}=[Q_{1},Q_{2}]=Q_{1}Q_{2}-Q_{2}Q_{1}$$

In SpinDynamica, the commutation superoperator of an <operator> is constructed as follows:

CommutationSuperoperator[<operator>]

Its properties are illustrated below:


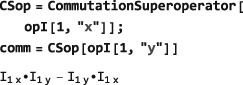


The SpinDynamica symbol ExpressOperator (see Section 6.4) may be used to evaluate the commutator.


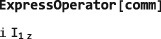


Complex exponentials of commutation superoperators are particularly important, since they represent spin evolution under a time‐independent Hamiltonian.(28, 29) When the complex exponential of a superoperator is applied to an operator, the result is a “sandwich” of the operator between two complex operator exponentials,(29) for example:


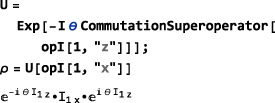



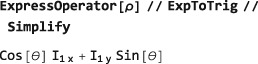


This example also shows how the SpinDynamica routine ExpressOperator and the Mathematica routines ExpToTrig and Simplify may be used to simplify the result.

### 5.2 **RotationSuperoperator**

Just as SpinDynamica uses RotationOperator for the complex exponentials of angular momentum operators, SpinDynamica uses RotationSuperoperator for the complex exponentials of the commutation superoperators of angular momentum operators. RotationSuperoperator uses identical syntax to RotationOperator, as follows:

$$\begin{array}{ccc} & \text{RotationSuperoperator[} & \\ & \text{<spins>,\{<angle>,<axis>\}}\\ & \text{]}\\ & \text{RotationSuperoperator[}\\ & \text{<spins>,\{<angle>,<phase>\}}\\ & \text{]}\\ & \text{RotationSuperoperator[}\\ & \text{<spins>,\{<angle>,\{<phase>,<tilt>\}\}}\\ & \text{]} \end{array}$$

It is possible to reproduce the results of the previous section by using RotationSuperoperator:


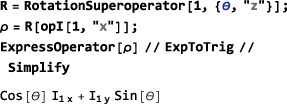


### 5.3 **DoubleCommutationSuperoperator**

Double‐commutation superoperators play a central role in spin relaxation theory.(28, 29, 30) They are given by the product of two commutation superoperators and may be defined in SpinDynamica by the symbol DoubleCommutationSuperoperator, which takes two operator arguments:

$$\begin{array}{ccc} & \text{DoubleCommutationSuperoperator[} & \\ & \text{<operatorA>,<operatorB>}\\ & \text{]} \end{array}$$

where <operatorA> and <operatorB> may differ.

When a double‐commutation superoperator is applied to an operator, the commutator with the second operator is taken before the commutator with the first operator, as shown by the the following example:


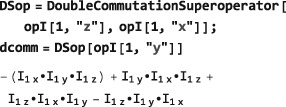


The double commutator is the result of first applying the ${\hat{I}}_{x}$ commutation superoperator and then the ${\hat{I}}_{z}$ to *I*_*y*_. The double commutator may be evaluated by using ExpressOperator.


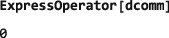


This result may be understood as follows. The first commutator amounts to:

(8) $$[I_{x},I_{y}]=iI_{z}$$

Forming the second commutator with the previous result leads to

(9) $$i[I_{z},I_{z}]=i0=0$$

which is the result returned by SpinDynamica.

### 5.4 **CoherenceOrderFiltrationSuperoperator**

Section 4.4.2 introduced the CoherenceOrder function, which returns the set of coherence orders for an arbitrary density operator. Many common pulse sequences eliminate signals passing through unwanted coherence orders by using phase cycling or pulsed field gradient procedures.(2, 28) The projection of an operator onto the subspace of desired coherence orders is implemented in SpinDynamica by the symbol CoherenceOrderFiltrationSuperoperator. The syntax takes the general form:

$$\begin{array}{ccc} & \text{CoherenceOrderFiltrationSuperoperator[} & \\ & \text{<spins>,<coherenceorders>}\\ & \text{]} \end{array}$$

The <spins> argument may be a single spin label or a list of spin labels. The <coherenceorders> argument may be a single coherence order, or a list of coherence orders. If the <spins> argument is omitted, all spins in the current SpinSystem are assumed. The example below shows the selection of zero‐quantum density operator components:


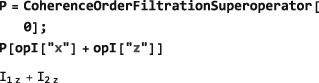


Operators may also be filtered through the coherence orders of a specific spin, or set of spins, for example:


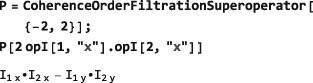


#### 5.4.1 **Superoperator**

The routine Superoperator allows the definition of a superoperator from its matrix representation in a Liouville space basis (see below), in similar fashion to the routine Operator, which acts in Hilbert space (see Section 4.3.5).

#### 5.4.2 More superoperators

SpinDynamica contains many other superoperator definitions, including

- UnitySuperoperator
- NullSuperoperator
- ProjectionSuperoperator
- ShiftSuperoperator
- SphericalComponentFiltrationSuperoperator
- SpinPermutationSuperoperator

Consult the SpinDynamica documentation for details.

## 6 OPERATOR BASES AND LIOUVILLE SPACE

### 6.1 **LiouvilleBracket**

The code in this section assumes prior execution of the command SetSpinSystem[1].

The *Liouville bracket* of two operators *A* and *B* is defined:

(10) $$\left(A|B)=\text{Tr}\{A^{†}B\right\}$$

where Tr denotes the trace of the matrix representation and the dagger denotes the *adjoint* (transpose of the complex conjugate).(29) Two operators are said to be *orthogonal* if their Liouville bracket is zero. The Liouville bracket may be computed in SpinDynamica by using the LiouvilleBracket symbol:


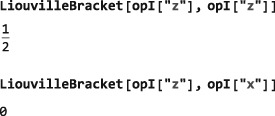


The Liouville bracket depends on the dimension of Hilbert space and hence the value of SpinSystem[]. The Liouville bracket of an operator with itself is equal to the square of its norm (Section 4.4.3).

### 6.2 Operator bases

The code in this section assumes prior execution of the command SetSpinSystem[2] and SetOperatorBasis[DefaultOperatorBasis[]].

A Liouville space is spanned by a set of $N_{L}=N_{H}^{2}$ orthonormal spin operators {*Q*_1_,*Q*_2_…}, where *N*_*H*_ is the dimension of Hilbert space (Equation 1). The orthonormality of the basis operators corresponds to the following equation:

(11) $$(Q_{j}|Q_{k})=\delta_{jk}$$

where *δ*_*jk*_ is equal to 1 if *j* and *k* are the same, but 0 otherwise. A set of *N*_*L*_ orthonormal operators comprises an *operator basis*.

SpinDynamica contains several predefined operator bases and allows users to define their own.

The current operator basis may be inspected by executing the command OperatorBasis[]. A particular operator basis may be set for subsequent calculations by executing a command of the form SetOperatorBasis[<operatorbasis>] where the <operatorbasis> argument may have one of the forms specified below.

#### 6.2.1 **ShiftAndZOperatorBasis**

The default operator basis for spin‐1/2 systems is the ShiftAndZOperatorBasis, which is given by all linear independent combinations of shift and z spin operators. In the case of two‐spin‐1/2 particles, the default operator basis is as follows:


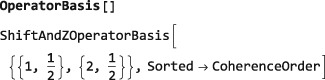
 which indicates that the default ShiftAndZOperatorBasis belongs to a system of two spins‐1/2 with labels 1 and 2, and that the basis operators are sorted according to their coherence order. The set of basis operators is revealed by executing BasisOperators[]:


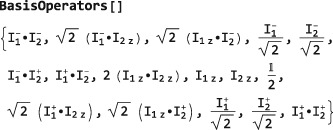


Note that all operators are normalized by suitable numerical coefficients.

The sorting of the basis operators according to coherence order may be revealed by mapping the CoherenceOrder symbol onto the BasisOperators[]:

CoherenceOrder/@BasisOperators[]

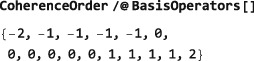


This example illustrates the use of the Mathematica Map routine (abbreviated /@), which applies a function to all members of a list. The result of the mapping is a list of the coherence orders for each operator and shows that the basis operators are ordered in ascending coherence order.

#### 6.2.2 **CartesianProductOperatorBasis**

Another useful operator basis is given by CartesianProductOperatorBasis, which consists of all combinations of products of the x‐, y‐ and z‐angular momentum operators:


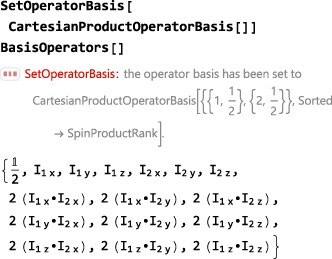


By default, the CartesianProductOperatorBasis is ordered in ascending spin product rank (i.e., numbers of operators in the operator product).

#### 6.2.3 SphericalTensorOperatorBasis

Irreducible spherical tensor operators are used extensively in NMR relaxation theory and solid‐state NMR.(30, 31, 32, 33) An operator basis consisting of spherical tensor operators may be set up as follows:

$$\begin{array}{ccc} & \text{SetOperatorBasis[} & \\ & \text{SphericalTensorOperatorBasis[]}\\ & \text{]} \end{array}$$

The basis operators for a SphericalTensorOperatorBasis in a two‐spin‐1/2 system are given by:


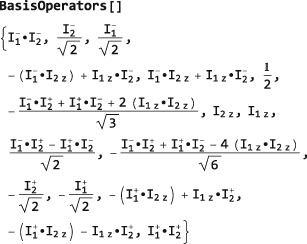


The STO basis operators have a sharp coherence order and transform in a controlled manner under rotations.

#### 6.2.4 Other operator bases

SpinDynamica contains several more preprogrammed operator bases, including

- ShiftAndPolarizationOperatorBasis
- ZeemanKetBraOperatorBasis
- SingletTripletOperatorBasis

Various predefined sort orders are available for some of these bases. In addition, user‐defined operator bases may be generated by deploying the DefineOperatorBasis routine. Consult the SpinDynamica documentation for details.

#### 6.2.5 **OperatorBasisDimension**

The number *N*_*L*_ of basis operators in a given Liouville basis may be determined by executing the command OperatorBasisDimension[<operatorbasis>], where as usual, the absence of the <operatorbasis> argument indicates the current operator basis.

### 6.3 **SuperoperatorMatrixRepresentation**

The code in this section assumes prior execution of the command SetSpinSystem[2].

The matrix representation of a superoperator in a given operator basis may be computed using the syntax:

$$\begin{array}{ccc} & \text{SuperoperatorMatrixRepresentation[} & \\ & \text{<superoperator>,<operator basis>}\\ & \text{]} \end{array}$$

The current operator basis is assumed if the second argument is omitted.

The matrix representation of a superoperator $\hat{S}$ in a given operator basis is a *N*_*L*_×*N*_*L*_‐dimensional square matrix, where *N*_*L*_ is the dimension of the Liouville space. The matrix elements are given by

(12) $$S_{jk}=(Q_{j}|\hat{S}|Q_{k})=\text{Tr}\{Q_{j}^{†}\hat{S}Q_{k}\}$$

and may be calculated individually using the LiouvilleBracket routine:


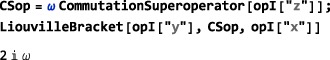


Superoperator matrix representations may be visualized in the same way as operator matrix representations. For example, the matrix representation of the commutation superoperator for the scalar coupling interaction **I**_1_·**I**_2_:


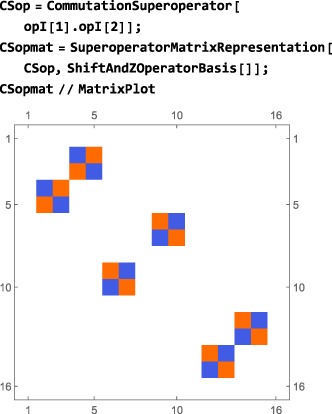


### 6.4 **ExpressOperator**

The code in this section assumes prior execution of the command SetSpinSystem[1].

A given operator may be expressed as a combination of basis operators by using ExpressOperator. This is often useful for simplifying the result of an operator calculation, or for transforming operators into a desired form. The general syntax is as follows:

$$\begin{array}{ccc} & \text{ExpressOperator[} & \\ & \text{<operator>,<operatorbasis>}\\ & \text{]} \end{array}$$

where the current OperatorBasis is assumed if <operatorbasis> is omitted.

The following examples show how Cartesian spin operators may be expressed as combinations of shift operators:


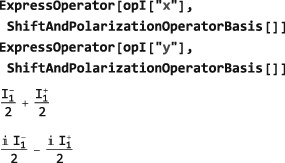


returning standard text books results. Similarly, a z‐angular momentum operator may be expressed in terms of polarization operators(28):


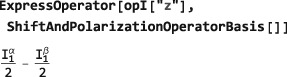
 which indicates that the z‐angular momentum operator measures the population difference between *α* and *β* states.

## 7 DENSITY OPERATORS

The code in this section assumes prior execution of the command SetSpinSystem[2].

The quantum state of the spin ensemble is described by the *spin density operator*, given by the operator:

(13) $$\rho =\bar{|\psi \rangle \langle \psi }|$$

where |*ψ*⟩ is the quantum state of a single spin system and the overbar denotes an average over all ensemble members.(2, 28) A density operator defined by Equation 13 has unit trace, Tr{*ρ*}=1.

Spin dynamical calculations often use a loose specification of the initial density operator, of the form opI[“z”], to indicate that the spin angular momentum is initially polarized along the z‐axis (conventionally, the main magnetic field direction). Subsets of z‐polarized spins may be specified using syntax of the form opI[1,“z”] and opI[{2,3},“z”], as discussed in Section 4.3.1. The practice of using unadorned angular momentum operators to represent spin density operators is widespread in NMR theory(2, 28) and is usually acceptable for calculations at ordinary temperatures on weakly polarized spin systems.

SpinDynamica allows any operator to be used for the spin density operator, even if it does not have unit trace. The routines presented below facilitate the construction of rigorous initial spin density operators.

### 7.1 **ThermalEquilibriumDensityOperator**

The density operator for a spin system in thermal equilibrium at temperature *T* in the presence of a laboratory frame Hamiltonian $H_{\text{lab}}$ may be written as follows:

(14) $$\rho_{\text{eq}}=\frac{\exp\{-\hbar H_{\text{lab}}/k_{B}T\}}{\text{Tr}(\exp\{-\hbar H_{\text{lab}}/k_{B}T\})}$$

This density operator may be generated in SpinDynamica by the syntax

(15) $$\begin{array}{c} \text{ThermalEquilibriumDensityOperator[}\;\text{<Hlab>}\;,\\ \text{<Temperature>,<options>]} \end{array}$$

where <Hlab> is the laboratory frame Hamiltonian and <Temperature> is the temperature (in Kelvin).

The <options> may include a setting for the HighTemperatureApproximation symbol. The default setting is HighTemperatureApproximation→True, in which case, a high‐temperature approximation is used to evaluate Equation 14:

(16) $$\rho_{\text{eq}}≃N_{H}^{-1}(1-\frac{\hbar H_{\text{lab}}}{k_{B}T})$$

where *N*_*H*_ is the Hilbert space dimension, as shown below:


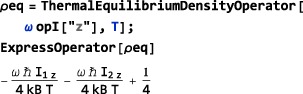


The high‐temperature approximation may be disabled by using HighTemperatureApproximation→False, as shown below:


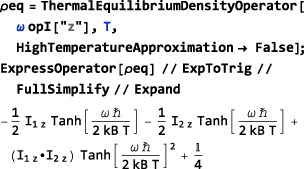


In the example above, the Mathematica routines ExpToTrig and FullSimplify are used to obtain a concise analytical form for the result.

### 7.2 **PolarizedDensityOperator**

The symbol PolarizedDensityOperator sets up a density operator with an arbitrary polarization level for one or more spins, along any axis. The syntax of PolarizedDensityOperator supports many special cases, but the most general form is given by:

$$\begin{array}{ccc} & \text{PolarizedDensityOperator[\{} & \\ & \text{\{<lab\#1>\{<p\#1>,<axis\#1>\}\},}\\ & \text{\{\{<lab\#2>\{<p\#2>,<axis\#2>\}\},…\}]} \end{array}$$

The polarization level of an individual spin, <p#j>, is bounded by −1 and 1, indicating parallel or antiparallel orientation with respect to the specified axis, <axis#j>.

Simplified syntax is available for special cases. For example, a density operator for an ensemble of spin‐1/2 pairs, with uniform polarization *p* along the z‐axis is constructed as follows:

PolarizedDensityOperator[p]

Generating the following result:


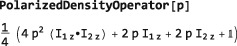


The quadratic terms are important at high polarization levels but may be neglected for thermally polarized spin systems.

Similarly, a density operator corresponding to individual polarization levels for different spins is specified as follows:


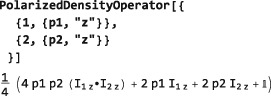


PolarizedDensityOperator may also be used to set up a density operator polarized along an arbitrary axis, by using the syntax:

$$\begin{array}{ccc} & \text{PolarizedDensityOperator[\{} & \\ & \text{\{<lab\#1>,\{<p\#1>,\{<tilt\#1>,<phase\#1>\}\}\},}\\ & \text{\{<lab\#2>,\{<p\#2>,\{<tilt\#2>,<phase\#2>\}\}\},...}\\ & \text{\}]} \end{array}$$

which leads to rather complex expressions in most instances.

In general, PolarizedDensityOperator leads to the following expression for the spin density operator: Consider an ensemble of systems each containing *N* spins. Suppose that the species *I*_1_ have a polarization *p*_1_ along the axis **n**_1_, the species *I*_2_ have a polarization *p*_2_ along the axis **n**_2_, and so on. The corresponding density operator has the form:

(17) $$\rho =N_{H}^{-1}\overset{N}{\underset{j=1}{\prod }}(1+2p_{j}I_{j}\cdot n_{j})$$

A special case of this equation is discussed in Eills et al.(20)

### 7.3 **SingletPolarizedDensityOperator**

This symbol sets up a spin density operator exhibiting an arbitrary degree of *singlet order* (population difference between the singlet and triplet states of spin‐1/2 pairs, see references herein.(19, 20, 27)). For example, the syntax SingletPolarizedDensityOperator[{{1,2},1}] generates a spin density operator exhibiting maximal singlet polarization for spin pairs {#1,#2}:


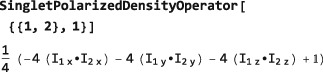


Initial density operators of this type are often used for the analysis of parahydrogen‐enhanced NMR phenomena.(20, 34, 35)

The general form of SingletPolarizedDensityOperator is defined as follows: Consider an ensemble of systems each containing *N* spins. Suppose that the species *I*_*j*_ and *I*_*k*_ are both spins‐1/2 and have a singlet polarization $p_{jk}^{S}$. The corresponding density operator has the form:

(18) $$\rho =N_{H}^{-1}(1-4p_{jk}^{S}I_{j}\cdot I_{k})$$

SingletPolarizedDensityOperator also allows the construction of a density operator in which distinct pairs of spins have different singlet polarization levels. Execute the instruction ?SingletPolarizedDensityOperator for details.

### 7.4 Derivation of polarization levels

SpinDynamica provides routines for deriving the degrees of polarization, which correspond to a given density operator. These routines should only be used for density operators defined in a “rigorous” way (i.e., deriving from ThermalEquilibriumDensityOperator, PolarizedDensityOperator, or SingletPolarizedDensityOperator.

#### 7.4.1 **PolarizationLevelOperator**

The symbol PolarizationLevelOperator may be used in combination with OperatorAmplitude(Section 4.4.1) to derive the degree of spin polarization implied by a given spin density operator.

For example, the following code sets the spin density operator to its thermal equilibrium value for protons in a magnetic field of 11.4 Tesla and 300 Kelvin and then determines the degree of polarization along the z‐axis under those conditions:


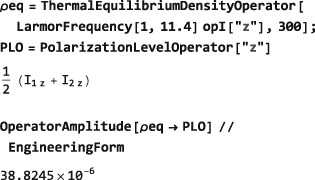


As expected, the degree of thermal polarization is very small.

The general form of PolarizationLevelOperator, for determining the mean polarization of a set of spins {*I*_*j*_,…} along the axis **n**, for an ensemble of *N*‐spin systems, is given by:

(19) $$PLO=3N_{H}^{-1}\sum_{j}{\left(I_{j}+1\right)}^{-1}I_{j}\cdot n$$

The symbol name PolarizationLevelOperator is used to avoid conflict with the term *polarization operator*, which is used by Ernst et al.(28) to denote the projection operators onto the spin‐1/2 Zeeman eigenstates, $I_{j}^{\alpha }$ and $I_{j}^{\beta }$.

#### 7.4.2 **SingletPolarizationLevelOperator**

The symbol SingletPolarizationLevelOperator behaves in a similar way to PolarizationLevelOperator but derives the degree of singlet order represented by a given density operator. The following example shows that highly polarized systems of spin‐1/2 pairs exhibit intrinsic negative singlet order (since the singlet state is depleted with respect to the lowest‐energy triplet state):


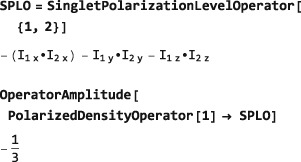


This phenomenon has been exploited experimentally.(36, 37, 38)

The general form of SingletPolarizationLevelOperator for the singlet polarization of spin‐1/2 pairs {*I*_*j*_,*I*_*k*_} in a *N*‐spin ensemble is given by:

(20) $$SPLO=-4N_{H}^{-1}I_{j}\cdot I_{k}$$

## 8 PROPAGATION

The central problem of spin dynamics is to propagate the state of the spin system (or spin ensemble) through a set of consecutive events in time.(39) For a single spin system described by a ket |*ψ*⟩(*t*) in Hilbert space, the equation of motion is given by the time‐dependent Schrödinger equation:

(21) $$\frac{d}{dt}|\psi \rangle (t)=-iH(t)|\psi \rangle (t)$$

where H is the Hamiltonian operator. In Liouville space, the quantum state is described by the density operator *ρ*(*t*), and the equation of motion is given by the Liouville‐von Neumann equation (LvN equation):

(22) $$\frac{d}{dt}\rho (t)=\hat{L}(t)\rho (t)$$

where $\hat{L}(t)$ is the *Liouvillian superoperator* of the system.

The Liouvillian may be time‐dependent and contain coherent or incoherent contributions.

(23) $$\hat{L}(t)={\hat{L}}_{\text{coh}}(t)+{\hat{L}}_{\text{incoh}}(t)$$

The coherent contributions refer to interactions that are identical for all members of the ensemble, while incoherent contributions derive from fluctuating Hamiltonians, which cause relaxation.

The coherent part of the Liouvillian is given by the commutation superoperator of the coherent Hamiltonian, multiplied by a complex factor:

(24) $${\hat{L}}_{\text{coh}}(t)=-i{\hat{H}}_{\text{coh}}(t)$$

The incoherent part of the Liouvillian is given (under suitable assumptions as to the timescale of the fluctuating interactions(29, 30)) by the *relaxation superoperator*,denoted $\hat{\Gamma }$:

(25) $${\hat{L}}_{\text{incoh}}(t)=\hat{\Gamma }(t)$$

### 8.1 Generators

In SpinDynamica, spin Hamiltonians H, Liouvillians $\hat{L}$, and relaxation superoperators $\hat{\Gamma }$, are known collectively as *generators*. These terms generate the evolution of the individual quantum states through the time‐dependent Schrödinger equation (Equation 21), or that of the ensemble spin density operator through the LvN equation (Equation 22).

#### 8.1.1 Null generator

A null generator is specified in SpinDynamica by the Mathematica symbol None.

#### 8.1.2 Spin Hamiltonians

The examples in this section assume prior execution of the command SetSpinSystem[2].

SpinDynamica syntax allows the use of both time‐independent and time‐dependent spin Hamiltonians.

1. *Time‐independent Hamiltonians*. A time‐independent spin Hamiltonian is given by a spin operator, defining the type of spin interaction, multiplied by a symbolic or numerical factor, which expresses the magnitude of the spin interaction.
  In SpinDynamica, the numerical multipliers of spin operators are always given in angular units (radians per second, rad s^−1^). For example, a chemical shift offset term, expressing a 10 Hz resonance offset, should be written as 2*π*10 opI[“z”]. Similarly, the interaction of spins with a radiofrequency field along the rotating frame x‐axis, with an amplitude corresponding to a 1 kHz nutation frequency, corresponds to a term of the form 2*π*10^3^opI[“x”]. A scalar interaction Hamiltonian could be defined symbolically as follows:

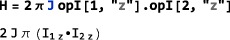

  Numerical values may be assigned to a symbolic coupling parameter by deploying Mathematica's replacement rules, for example:

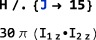

2. *Time‐dependent Hamiltonians*. Time‐dependent spin Hamiltonians are supported by SpinDynamica. The form of time‐dependence may be specified in several different ways:
  - *Arbitrary time‐dependence*. A Hamiltonian with a completely arbitrary time‐dependence may be specified by using the Function routine of Mathematica. Consider, for example, an amplitude‐modulated cosine pulse. The generator for such a pulse could be defined by using the following syntax:

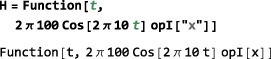

    This implies that the nutation frequency under the rf field is given by 100 Hz at the maximum in rf amplitude, and that the rf field undergoes a 10 Hz cosine modulation. Modulations of arbitrary form may be generated using the extensive library of analytical functions built into Mathematica.
  - *Periodic time‐dependence*. In the example above, the time‐dependent Hamiltonian term is periodic, meaning that it repeats itself at regular intervals (in this case, every 100 ms). In some cases, the periodicity of a time‐dependent spin Hamiltonian may be used to speed up numerical calculations(39, 40, 41, 42) or to derive analytical results. The entire field of Floquet theory in NMR is based on this insight.(43) SpinDynamica uses the symbol PeriodicFunction to specify time‐periodic modulations. The syntax of PeriodicFunction is given by
    $$\begin{array}{ccc} & \text{PeriodicFunction[} & \\ & \text{<var>,<period>,<function>}\\ & \text{]} \end{array}$$
    where the <period> argument may be used by SpinDynamica to deploy special numerical algorithms for periodic generators (it is up to the user to specify this period correctly; SpinDynamica cannot deduce the period automatically). The example of a cosine‐modulated pulse may be written using PeriodicFunction as follows:

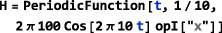

  - *Local time‐dependence*. The symbol t in the examples above refers to a *global time variable* that increases monotonically through the sequence of events. When this is not appropriate, SpinDynamica also allows the use of *local time variables*, as discussed in Section 8.3.2.

#### 8.1.3 Relaxation superoperators

Spin relaxation is incorporated into spin dynamical calculations by including a relaxation superoperator $\hat{\Gamma }$ as the incoherent part of the Liouvillian. The relaxation superoperator may either be *phenomenological*(meaning that one simply specifies some experimental observables, such as the relaxation times *T*_1_, *T*_2_, etc.) or may be based on detailed theoretical expressions for the relaxation mechanisms. SpinDynamica allows either, or both, of these methods to be utilized in calculations.

1. *Phenomenological relaxation*. Despite its mouthful of a name, the symbol PhenomenologicalRelaxationSuperoperator is convenient for incorporating simple relaxation terms into the spin dynamical evolution without worrying about mechanistic details. Its general syntax is given by
  $$\begin{array}{ccc} & \;\text{PhenomenologicalRelaxationSuperoperator[} & \\ & \;\text{\{\{<lab\#1>,<T1\#1>,<T2\#1>\},}\\ & \;\text{\{<lab\#2>,<T1\#2>,<T2\#2>\},…\}}\\ & \;\text{]} \end{array}$$
  which allows separate *T*_1_ and *T*_2_ values to be assigned to each type of spin in the ensemble. The simplified syntax PhenomenologicalRelaxationSuperoperator[T1,T2] assumes that all spins have the same *T*_1_ value, and also the same *T*_2_ value. The even simpler syntax PhenomenologicalRelaxationSuperoperator[T] assumes that all spins in the system have the same *T*_1_=*T*_2_=*T*. For an ensemble of single‐spin‐1/2 particles with the following basis specifications:

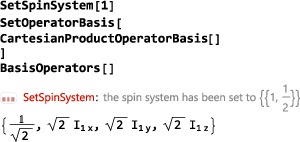

  The phenomenological relaxation superoperator takes the form:

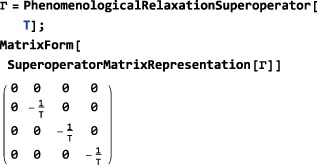
 which shows that density operator components proportional to *I*_*x*_, *I*_*y*_, and *I*_*z*_ all relax with the same time constant *T*.
  In the case of multiple‐spin systems, PhenomenologicalRelaxationSuperoperator assumes an uncorrelated relaxation model. For example, the Cartesian product operator term 4*I*_1*x*_ *I*_2*y*_ *I*_3*z*_ is assigned a relaxation rate constant equal to the sum of $T_{2}^{-1}$ for spins of type #1 and #2, added to $T_{1}^{-1}$ for spins of type #3.
2. *Mechanistic relaxation*. SpinDynamica allows the detailed specification of relaxation superoperators for arbitrary microscopic mechanisms.(30, 31) Such relaxation superoperators often consist of sums of DoubleCommutationSuperoperator symbols. Cross‐correlations, internal motions, and other exotic effects may all be treated.
  As a simple example of a mechanistic relaxation superoperator, consider relaxation of a one‐spin‐1/2 ensemble by fluctuating random fields along the z‐axis, with correlation time *τ*_*c*_. The corresponding relaxation superoperator has the form:
  (26) $$\hat{\Gamma }=-\gamma^{2}B_{z}^{2}\tau_{c}[{\hat{I}}_{z},{\hat{I}}_{z}]$$
  where *B*_*z*_ is the root‐mean‐square amplitude of the fluctuating field along the z‐axis, and *γ* is the gyromagnetic ratio. In SpinDynamica, this superoperator may be written as follows:
  $$\begin{array}{ccc} & \text{G=‐}\gamma^{\text{2}}{\text{B}}_{\text{z}}^{\text{2}}\tau_{c}\text{DoubleCommutationSuperoperator[} & \\ & \text{opI[“z”],opI[“z”]}\\ & \text{]} \end{array}$$
  The matrix representation of this superoperator in the CartesianProductOperatorBasis is given by:

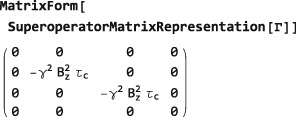

  The mechanistic relaxation superoperator is diagonal in this basis and exhibits two nonzero values. This indicates that the transverse angular momentum operators are exponentially damped with a rate constant equal to $\gamma^{2}B_{z}^{2}\tau_{c}$:
  (27) $$T_{2}^{-1}=\gamma^{2}B_{z}^{2}\tau_{c}$$
  while the longitudinal angular momentum *I*_*z*_ does not relax in this model.
  The reader is referred to the SpinDynamica documentation for more examples, including cross‐relaxation and nuclear Overhauser effects. For a sophisticated application involving internal rotation and cross‐correlation, see the studies of quantum rotor polarization effects in rotating methyl groups and methyl long‐lived states by Dumez et al.,(15) as well as long‐lived spin states in monodeuterated methyl groups by Elliott et al.(44)

Additional topics pertaining to relaxation superoperators are as follows:

1. *Secularization.* Relaxation Superoperators may be “secularized” in order to take into account the truncating effects of high magnetic field. The SpinDynamica routine Secularize accomplishes this task (both for operators and for superoperators).
2. *Thermalization.* Relaxation superoperators may be corrected for the finite temperature of the thermal environment. This is often necessary since long‐term evolution under an “untreated” relaxation superoperator leads to a spin system with zero order (corresponding to infinite temperature) rather than finite order (thermal equilibrium). The “standard” way to address this problem is to include the thermal equilibrium density operator *ρ*_eq_ in the relaxation part of the LvN equation, which then ceases to be a homogeneous first‐order differential equation.(28) SpinDynamica adopts an alternative method, which is more mathematically convenient since the homogeneous form of the LvN equation is retained.(29, 45) In this approach, the relaxation superoperator $\hat{\Gamma }$ is thermalized to ensure that it gives rise to the correct thermal equilibrium density operator, that is:
  (28) $${\hat{\Gamma }}^{\prime }\rho_{\text{eq}}=0$$
  where ${\hat{\Gamma }}^{\prime }$ is the thermalized relaxation superoperator and *ρ*_eq_ is the thermal equilibrium spin density operator, corresponding to the applied magnetic field and the temperature of the sample (Section 7.1). Thermalized relaxation superoperators have been used to predict and analyze a variety of magnetic resonance effects.(29, 45, 46, 47, 48, 49, 50) The ThermalizeSuperoperator routine may be used to adjust relaxation superoperators so that they satisfy Equation 28 and give the correct thermal equilibrium position for the spin density operator. An example is given below (Section 9.4.3). The SpinDynamica documentation provides some more examples.
  The current release of SpinDynamica (version 3.0.1) incorporates an accelerated procedure for the calculation of evolution under thermalized relaxation superoperators, as first implemented in GAMMA by Levante et al.(47)
3. *Time‐dependence.* Time‐dependent relaxation superoperators may be specified by using the routines Function and PeriodicFunction, in the same way as for Hamiltonians.

#### 8.1.4 **CombineGenerators**

The examples in this section assume prior execution of the command SetSpinSystem[1].

Any number of generators may be combined by using the routine CombineGenerators. The output of CombineGenerators takes into account any time‐dependence or periodicity and also converts a Hamiltonian into a Liouvillian generator, if the context requires. The general syntax is:

(29) CombineGenerators[<genA>,<genB>...]

Combining any generator with the null generator None leaves the original generator unchanged.

As a simple example consider combining a phenomenological relaxation superoperator with a chemical shift offset generator. This may be done in the following way:


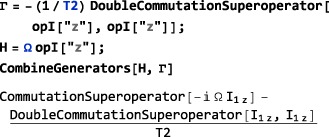


In order to allow combination with a relaxation superoperator, the Hamiltonian is expressed as a generator in Liouville space, using multiplication by the complex factor (−i), and conversion into a commutation superoperator.

In most cases, CombineGenerators handles the theoretical technicalities of combining generators without user intervention.

### 8.2 Numerical propagation: **NPropagate**

The central SpinDynamica routine for numerical solutions of the LvN equation is called NPropagate. The “N” of NPropagate refers to numeric, following Mathematica's naming conventions.

Most users of SpinDynamica should not need to use NPropagate explicitly, since the calls to NPropagate are handled by the top‐level routines described in Section 9.

The general syntax of NPropagate is as follows:

NPropagate[<events>,<options>][<rhoini>]

The <rhoini> argument specifies the initial value of the spin density operator. This may be any operator, including the rigorous density operator definitions discussed in Section 7.

The <events> argument specifies that the list of events over with the propagation is calculated. The syntax for this argument is discussed in Section 8.3.

The options argument consists of a sequence of replacement rules of the form <parameter #1>→<value #1>, <parameter #2>→<value #2>, etc., which specify the values taken by the optional parameters of the NPropagate routine. These rules may be specified in any order. Some important optional parameters are as follows:

1. InitialTimePoint and FinalTimePoint. These parameters set the origin of the global time variable used for the propagation (see Section 8.3.2). If these options are omitted, the default instructions apply, which are InitialTimePoint→0 and FinalTimePoint→Automatic. This implies that the global time variable has a value *t*=0 at the beginning of the sequence of events, with the final time point calculated automatically from the duration of the <events> argument. Similarly, the options InitialTimePoint→Automatic and FinalTimePoint→0 set the origin of the global time variable to the end of the sequence, with the (negative) initial time point calculated automatically from the duration of the <events> argument. NPropagate detects timing conflicts, in the case that both InitialTimePoint and FinalTimePoint are specified explicitly.
2. BackgroundGenerator. This option specifies a generator that acts throughout the sequence of <events>. Typically, this option is used to specify an “internal” spin Hamiltonian, or relaxation superoperator (or a combination of both), which acts at the same as the “external” interactions specified by the <events> argument. For example, the option setting BackgroundGenerator→2*π*100 opI[“z”] would cause a 100Hz resonance offset term to act throughout the calculation of the spin propagation. The value of BackgroundGenerator may be any valid generator, including time‐dependent generators specified using Function or PeriodicFunction (see Section 8.1.2), as well as combinations of generators constructed by using CombineGenerators (see Section 8.1.4). The default setting is BackgroundGenerator→None, indicating the absence of a background generator.
3. NDSolve options. In the case of time‐dependent generators, SpinDynamica typically exploits the Mathematica routine NDSolve for the numerical propagation of the spin density operator. NDSolve contains an extensive set of routines for the numerical solution of differential equations and may be controlled by a large set of option settings, as described in the Mathematicadocumentation. Appropriate option settings may be “passed down” to NDSolve by including them in the <options> argument to NPropagate. In most cases, this is not necessary, since the default settings work fine.
4. The Continuous option. The NPropagate routine may either return the final result of the spin dynamical propagation (Continuous→False), or the full trajectory of the density operator throughout the sequence of events (Continuous→True). The examples discussed here all employ the default setting Continuous→False. The option Continuous→True is deployed when NPropagate is called by the top‐level Trajectory routine (Section 9.4).

### 8.3 Events

**Figure 5:**
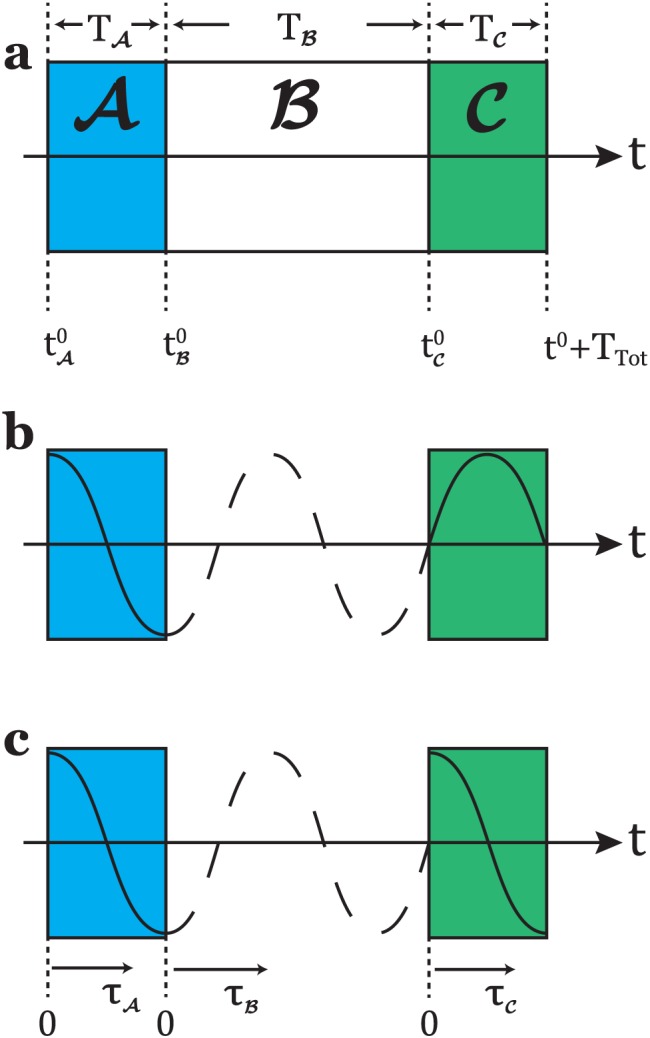
(a) An event sequence consisting of three elements, showing the initial time points and the global time variable; (b) the first and the last pulses use a rf field with a phase‐coherent cosine modulation. This is coded in SpinDynamica by using Function or PeriodicFunction with a global time variable (Equation 36); (c) the first and last pulses have an identical shape, but the modulation is not phase‐coherent. This is coded in SpinDynamica by using ShapeFunction with a local time variable (Equation 37)

We now consider the <events> argument to the <NPropagate> routine (Section 8.2).

f through a series of consecutive events {A,B,C⋯} with durations {*T*_*A*_,*T*_*B*_,*T*_*C*_…}(see Figure 5). In SpinDynamica, a chronological sequence of events is written as a conventional Mathematica list, that is,

(30) <events>={A,B,C⋯}

which should be read from left to right in chronological order.

#### 8.3.1 Event types

There are two types of events: *Instantaneous Events*, and *Finite Events*. Instantaneous events imply an immediate transformation of the spin density operator, with no elapsed time, while finite events concern evolution of the spin density operator under one or more generators, for a finite amount of time.

1. *Instantaneous events.* This type of event is specified by including a superoperator in the event list. For example, an event list of the form:
  (31) $$\begin{array}{ccc} & \text{<events>}=\{A,B, & \\ & \;\text{RotationSuperoperator}[\{\pi /2,\text{“x”}\}],\\ & \;\;\;D\text{⋯}\} \end{array}$$
  evolves the spin density operator through events A and B, before applying an instantaneous rotation using the specified RotationSuperoperator, and then continuing to evolve through D, and so on.
  Any superoperator may be used to specify an instantaneous event. The most common superoperators used for this purpose are RotationSuperoperator and CoherenceOrderFiltrationSuperoperator. The action of idealized, infinitely short radiofrequency pulses may be represented by using RotationSuperoperator, while the action of idealized phase cycling or field‐gradient coherence order selection schemes may be represented by using CoherenceOrderFiltrationSuperoperator.
2. *Finite events.* A finite event is specified by a generator‐duration pair, as follows:
  {<generator>,<duration>}
  where <generator> is the generator for event E, and <duration> is its duration. Numerical values for a duration are given in units of seconds.
NPropagate computes the spin evolution under a finite event by first combining the local event generator <generator> with the value of BackgroundGenerator (Section 8.2), using the CombineGenerators routine (Section 8.1.4). If the combined generator is time‐dependent, the Mathematica routine NDSolve routine is used to compute the evolution, either in Hilbert space (for Hamiltonians) or in Liouville space (for Liouvillians). In the case of time‐independent generators, the internal SpinDynamica routines typically use the matrix exponential MatExp routine of Mathematica.
  As an example, the finite event specified below indicates the interaction with an rf field along the rotating frame x‐axis, with an amplitude corresponding to a nutation frequency of 100 Hz, for an interval of 5 ms:
  $$\left\{\text{2}\pi \;\text{100}\;\text{opI[“x”]},\text{5}\times {\text{10}}^{-3}\right\}$$
  If the background generator takes its default value BackgroundGenerator→None, this event would lead to a *π* rotation of the spins around the rotating frame x‐axis. However, if the BackgroundGenerator option of NPropagate specifies a nontrivial generator, the evolution under this finite event depends on the combined action of BackgroundGenerator and the local event generator (given by 2*π*100 opI[“x”] in the case above.
  Event syntax of the form {None,T} may be used to specify a “delay” of duration T, during which, the BackgroundGenerator acts alone. If the local <generator> and BackgroundGenerator are both equal to None, there is no spin evolution at all.

#### 8.3.2 Time variables

SpinDynamica allows time‐dependent generators to be specified using either *global* or *local* time variables. The global time variable extends over the entire sequence of events, while local time variables are specific to each event. Each is useful in different circumstances.

1. *Global time variable.* Consider the evolution of a spin ensemble through a series of events <events> ={A,B⋯} with durations {*T*_*A*_,*T*_*B*_…}(see Figure 5). Suppose that the sequence of events starts at time point *t*_0_, such that the starting time points of the individual events are given by
  (32) $$\begin{array}{ccc} t_{A}^{0} & =t_{0} & \\ t_{B}^{0} & =t_{A}^{0}+T_{A}\\ t_{C}^{0} & =t_{B}^{0}+T_{B} \end{array}$$
  and so on. The starting time point of any event in the sequence may be determined recursively from the starting time point of the entire sequence *t*_0_, assuming that all the event durations are known. Denote the total sequence duration by *T*_tot_.
  The value of *t*_0_ is given by the InitialTimePoint and FinalTimePoint options for NPropagate (Section 8.2). For example, calling NPropagate with the default options InitialTimePoint→0 and FinalTimePoint→Automatic sets the time origin to *t*_0_=0. Calling NPropagate with the options InitialTimePoint→Automatic and FinalTimePoint→0 sets the time origin to *t*_0_=−*T*_tot_.
  The global time variable *t* increases monotonically, from *t*_0_ at the beginning of the sequence to *t*_0_+*T*_tot_ at the end of the sequence. Time‐dependent generators may be written in terms of the global time variable *t* by using the Function and PeriodicFunction symbols, as explained in Section 8.1.2.
  A time‐dependent generator is usually written in terms of a global time variable when the underlying physical process is independent of the applied pulse sequence. This is the case, for example, in magic‐angle spinning NMR, where the modulation of the spin Hamiltonian by the sample rotation is independent of the applied rf fields. The use of PeriodicFunction is appropriate in this case.
2. *Local time variables.* A local time variable, denoted *τ*_*A*_, may be defined for an event A, as follows:
  (33) $$\tau_{A}=t-(t_{A}^{0}+x_{A}T_{A})$$
  where *x*_*A*_ is a number between 0 and 1. The value of *x*_*A*_ determines the position of the local time origin: If *x*_*A*_=0, the local time origin *τ*_*A*_=0 is at the start of the event; if *x*_*A*_=1, the local time origin *τ*_*A*_=0 is at the end of the event; if *x*_*A*_=1/2, the local time origin *τ*_*A*_=0 is at the center point of the event.
  The local time variable *τ*_*A*_ increases monotonically from *τ*_*A*_=−*x*_*A*_ *T*_*A*_ at the beginning of the event A, through *τ*_*A*_=0 at the local time origin, to *τ*_*A*_=(1−*x*_*A*_)*T*_*A*_ at the end of the event.
  The SpinDynamica routine ShapeFunction allows the time‐dependence of a generator to be specified using one or more local time variables. The syntax for a single local time variable has the form:
  (34) ShapeFunction[{τ,x},<gen>]
  where the generator <gen> depends on the local time variable *τ*, and the parameter *x* governs the position of the local time origin, relative to the event itself.
  For example, a Gaussian‐shaped radiofrequency pulse may be expressed using the following event specification:
  (35) $$\begin{array}{ccc} \text{<event>} & =\{\text{ShapeFunction}[\{\tau ,1/2\}, & \\ & \omega_{\max}\text{Exp}[-\tau^{2}/2\sigma^{2}]\text{opI[“x”]],T}\} \end{array}$$
  where *ω*_max_ is the nutation frequency at the maximum rf field, the variance *σ* ^2^ determines the width of the Gaussian pulse in time, and *T* is its overall duration. The use of *x*=1/2 ensures that the maximum of the Gaussian shape is at the midpoint of the pulse event.
  Note that it would not be possible to express a shaped pulse reliably using a global time variable, since the value of the global time variable depends on the position of the pulse in the sequence of events. The shape of a radiofrequency pulse is local to the event itself and should be independent of the position of the pulse in the sequence.

The difference between global and local time variables is illustrated in Figure 5 (parts b and c). In both cases, two pulses employing a cosine‐modulated rf field are applied (events A and C). They are separated by a null event B of finite duration, *T*_*B*_. In case (b), the cosine modulations of the two pulses are phase‐coherent with respect to a global time variable. In case (c), the two pulses have the same internal waveform but are not phase‐coherent with each other.

Case (b) may be encoded as follows:

(36) $$\begin{array}{ccc} & \text{<events>}=\{ & \\ & \{\text{Function}[\text{t},\omega_{\max}\text{Cos}[\omega_{\text{mod}}\text{t}]]\text{opI[“x”]},T_{A}\},\\ & \text{\{None},T_{B}\},\\ & \left\{\text{Function[t,}\omega_{\max}\text{Cos}[\omega_{\text{mod}}\text{t]]opI[“x”]},T_{C}\right\}\\ & \} \end{array}$$

where *ω*_max_ is the nutation frequency at the pulse maximum, *ω*_mod_ is the modulation frequency, and *T* is the duration of both pulses A and C. The use of Function with a global time variable ensures that the modulations of both pulses are phase‐coherence, independent of the intervening element B.

Case (c) may be encoded as follows:

(37) $$\begin{array}{ccc} & \text{<events>}=\{ & \\ & \{\text{ShapeFunction}[\{\tau ,\text{0}\},\omega_{\max}\text{Cos}[\omega_{\text{mod}}\tau ]]\text{opI[“x”]},T_{A}\},\\ & \{\text{None},T_{B}\},\\ & \left\{\text{ShapeFunction}[\{\tau ,\text{0}\},\omega_{\max}\text{Cos}[\omega_{\text{mod}}\tau ]]\text{opI[“x”]},T_{C}\right\}\\ & \} \end{array}$$

The use of a local time variable *τ*, with a time origin starting at the beginning of each event (*x*=0), ensures that both pulses have an identical shape, starting at the maximum of the cosine function.

The ShapeFunction routine of SpinDynamica also supports a more general syntax, allowing a generator to be written in terms of the global time variable as well as several local time variables, with the option of indicating periodicity. Details are made available by executing ?ShapeFunction.

#### 8.3.3 PulseSequence objects

SpinDynamica contains a set of symbols that facilitate the construction of event sequences for the interactions of spins with applied rf fields, in the rotating reference frame. The intention is to mimic a typical pulse‐programming environment on an NMR spectrometer, including symbols such as Pulse, Delay, and ShapedPulse.

The currently existing functionality is as follows:

- Pulse
  The Pulse symbol generates a SpinDynamica event for the interaction of the spins with a rectangular rf pulse, expressed as a rotating‐frame Hamiltonian. The flip angle, phase, nutation frequency, and duration of the pulse may be specified (conflicts between these specifications generate a warning). The general syntax is
  $$\begin{array}{c} \text{Pulse[<labels>,\{<flip angle>,<phase>\},}\\ \text{<options>]} \end{array}$$
  where the <labels> argument indicates the spin or spins that interact with the rf field. All spins are assumed to interact, in the case that <labels> is missing.
  For example, the syntax
  Pulse[{π,“y”}]
  generates an ideal pulse of flip angle *ϕ* and phase *π*/2 (corresponding to the SpinDynamica event RotationSuperoperator[{*π*,“y”}]). The syntax
  $$\text{Pulse}[\{\pi ,\text{“y”}\},\text{PulseDuration}\;\to \;10\times 10^{-6}]$$
  generates the SpinDynamica event for the interaction with a rectangular pulse of duration 10*μ*s. Since this implies a nutation frequency of 50 kHz, the corresponding SpinDynamica event is:
  $$\left\{2\pi \;50\times 10^{3}\;\text{opI[“y”]},10\times 10^{-6}\right\}$$
  Similarly, the syntax
  $$\begin{array}{ccc} & \text{Pulse}[\{\pi ,\text{“y”}\}, & \\ & \;\;\text{NutationFrequency}\;\to \;2\pi \;10\times 10^{3}] \end{array}$$
  generates an event corresponding to the interaction of the spins with an rf field, whose amplitude corresponds to a nutation frequency of 10 kHz, and with the pulse duration calculated automatically (50 *μ*s in this case).
  The option AmplitudeFactor scales the rf amplitude while keeping the pulse duration constant. This feature may be used to simulate the effect of rf inhomogeneity. The default value AmplitudeFactor→1 corresponds to the nominal amplitude, while AmplitudeFactor→0.9 indicates an rf field, which is 10% lower than nominal.
- Delay
  The syntax Delay[<duration>] generates a SpinDynamica event of the form {None,<duration>}.
- ShapedPulse
  This routine generates a SpinDynamica event corresponding to the interaction of the spins with a rf pulse of arbitrary waveform. Analytical forms for the amplitude, phase, and frequency may all be specified. The general syntax is
  $$\begin{array}{c} \text{ShapedPulse[<labels>,<duration>,}\\ \left\{\tau ,\text{<waveform>}]\},\text{<options>}\right] \end{array}$$
  where <labels> indicates the set of interacting spins (omission indicates that all spins interact with the field), <duration> indicates the duration of the shaped pulse in seconds, *τ* is a local time variable (see Section 8.3.2), and <waveform> defines the shape of the pulse. In general, the <waveform> argument has the form
  {<amplitude>,<phase>,<frequency>}
  where <amplitude>, <phase>, and <frequency> may all be functions of the local time variable *τ*. The <options> argument may contain a rule for AmplitudeFactor, which plays the same role as for Pulse (see above).
  The translated form of a ShapedPulse event has the form {ShapeFunction[…],<duration>}. An example of ShapedPulse is given in Section 9.4.2.
- PulseSequence
  The PulseSequence symbol may be used to invest a list of Pulse, Delay, and ShapedPulse objects with a set of global settings for NutationFrequency, AmplitudeFactor, etc., as well as an overall phase shift. Execute ?PulseSequence for details.

The pulse sequence functionality of SpinDynamica is in development at the time of writing. Most of the examples below use explicit specification of the Hamiltonians and durations.

#### 8.3.4 More event routines

SpinDynamica contains additional routines for the generation and analysis of event sequences. These include

- EventDuration
- Repeat
- Precalculate

Consult the SpinDynamica documentation for details.

### 8.4 Propagation examples

The code in this section assumes prior execution of the command SetSpinSystem[2] and SetOperatorBasis[CartesianProductOperatorBasis[]].

#### 8.4.1 The spin echo

As an illustrative example consider the effect of a spin‐echo sequence on a single‐spin‐1/2 ensemble, in the presence of a resonance offset. The spin‐echo sequence is specified as follows, assuming two delays of 0.5 s each, bracketing an ideal *π*_*x*_ pulse:


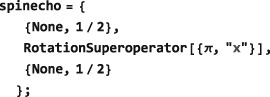


Assuming the initial magnetization to be aligned along the x‐axis, the response of the system is given by:


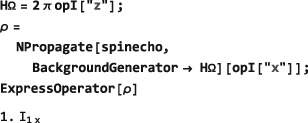


This shows that the spin‐echo block refocuses the evolution of the system. The use of ExpressOperator ensures a simplified output of the final density operator.

#### 8.4.2 INEPT

A commonly employed polarization transfer scheme in solution‐state NMR is the INEPT sequence.(2, 51) In a two‐spin‐1/2 ensemble, the polarization of spin 1 is transfered to spin 2 through secular J‐couplings. The Hamiltonian of such a system is given by:


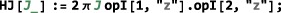


The corresponding pulse sequence for such a system might be defined as shown below:


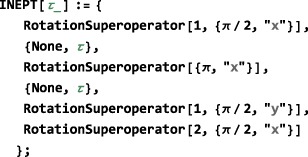


The polarization transfer between the pair of spins is maximized if the duration of free evolution periods equals *τ*=1/(4*J*). Choosing a J‐coupling strength of 15 Hz NPropagate returns the following result:


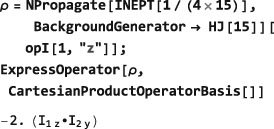


Where the product operator indicates pure antiphase magnetization on spin 2.

### 8.5 Symbolic propagation

At the time of writing, SpinDynamica does not have a fully general routine for symbolic propagation. Nevertheless, it is possible to perform symbolic calculations with a small amount of Mathematica programming. The routines Propagate and Evolve are defined by executing the following lines:

The first definition of Propagate is responsible for the propagation of finite events, while the second definition handles instantaneous events. Propagation through a list of events is performed by the Evolve routine.

These routines enable the analysis of solution‐state NMR sequences by the product operator formalism.(2, 28) As an example, consider the INEPT sequence again. Using symbolic values for the coupling strength (*J*) and the evolution delay (*τ*), analytic results may be derived by making use of the Evolve routine.


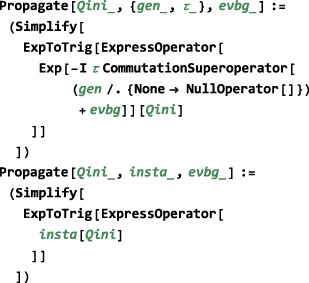



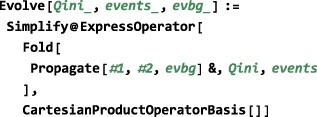



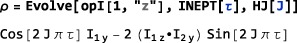


When *τ* is set equal to 1/(4*J*), the previous result is retrieved:


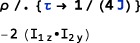


## 9 SIMULATIONS

The examples in this section assume prior execution of the command SetSpinSystem[2].

### 9.1 Syntax

SpinDynamica provides a small number of top‐level simulation routines that deploy NPropagate and the underlying machinery in order to perform common spin dynamical calculation tasks with a minimum of user programming. All of these routines use standard syntax of the form <toplevelroutine>[<arguments>,<options>], where the syntax of <arguments> is specific to the routine, as described below, and <options> is a sequence of replacement rules.

#### 9.1.1 General options

Some of the option settings for a top‐level routine are specific to the top‐level routine, as described below. Other options are passed down to the NPropagate routine, which performs the spin dynamical propagation. An important example is the setting for BackgroundGenerator, which is passed from the top‐level routine to NPropagate. This allows specification of the internal spin Hamiltonian and/or relaxation superoperator.

Settings for NDSolve may also be specified at the top level; these are first passed to NPropagate, which passes them down further to the Mathematica routine NDSolve, as described in Section 8.2.

#### 9.1.2 **EnsembleAverage**

All top level routines may provide a value for the EnsembleAverage symbol, by including an option setting of the form

EnsembleAverage→{<var>,<samplingscheme>}

This works as follows: Suppose that <arguments> or other <options> settings of the top‐level routine contain expressions that depend on the symbolic variable <var>. The EnsembleAverage instruction causes the calculation to be repeated many times, with the value of <var> taking the values specified in <samplingscheme>, and the results combined when all calculations are complete.

There are several syntactical possibilities for the <samplingscheme>. The simplest syntax for <samplingscheme> takes the form

{<val#1>,<val#2>...}

In this case, the variable <var> takes the indicated set of values, with all simulations combined with equal weights.

It is also possible to specify the weights explicitly:

{{val#1,weight#1},{val#2,weight#2},...}

More advanced constructions may be used, which allow several independent variables to be used in the EnsembleAverage and for the ensemble average values to be given by lists of several elements. Further details may be found in the SpinDynamica documentation.

There are preprogrammed sampling schemes for special purposes; the SpinDynamica symbol OrientationalSamplingScheme allows access to a number of specialized schemes for the powder averaging of orientational Euler angles in solid‐state NMR.(26, 52) Samples of general multivariate distributions may be generated by deploying Mathematica routines such as RandomVariate. A simple example is given in Section 9.5.8.

The setting for the option Parallel controls whether the individual calculations of an EnsembleAverage are performed in sequence on one processor (Parallel→False) or in parallel on all the processors, which are accessible to the Mathematica kernel (Parallel→True). The default setting for the top‐level routines is Parallel→True.

#### 9.1.3 Initial and observable operators

Each simulation routine allows specification of the initial density operator and one or more observable operators. SpinDynamica allows any operator to be used for this purpose.

Rigorously defined initial density operators are made available through the ThermalEquilibriumDensityOperator, PolarizedDensityOperator, and SingletPolarizedDensityOperator routines.

Any spin operator may be used as an observable; the polarization level of any subset of spins, in any desired direction, may be probed by using the PolarizationLevelOperator as an observable (see Section 7.4.1). The singlet polarization level of spin pairs may be probed by using SingletPolarizationLevelOperator as an observable (see Section 7.4.2).

#### 9.1.4 **NormalizationFactor**

All simulation routines support the optional argument NormalizationFactor→<normfactor>. This causes the calculated results to be divided by <normfactor>.

### 9.2 **TransformationAmplitude**

A frequent aim of a pulse sequence is to transform some initial density operator into a desired final density operator. The efficiency of the transformation is given by the overlap of the final density operator and the target operator. This may be calculated by the top‐level routine TransformationAmplitude. In its simplest form, this takes the following syntax:

$$\begin{array}{ccc} & \text{TransformationAmplitude[} & \\ & \text{<rhoini>}\to \text{<Qobs>,<events>,<opts>}\\ & \text{]} \end{array}$$

<rhoini> symbolises the initial density operator and <Qobs> the target operator. The right arrow ( →) indicates that <rhoini> evolves under the sequence of events and is then projected onto <Qobs>.

The initial density operator <rhoini> may be any operator, including the operators generated by the symbols ThermalEquilibriumDensityOperator, PolarizedDensityOperator, and SingletPolarizedDensityOperator, as described in Section 7.

The target operator <Qobs> may be any operator.

If the initial density operator is defined rigorously, and PolarizationLevelOperator is used as a target operator (Section 7.4.1), then TransformationAmplitude returns the specified polarization level at the end of the sequence of events. Similarly, the singlet polarization level at the end of the sequence is obtained by using SingletPolarizationLevelOperator as a target operator (see Sections 7.4.1 and 7.4.2).

TransformationAmplitude may be used to calculate the transformation amplitudes of an initial density operator into several target operators at the same time by using the syntax:

$$\begin{array}{ccc} & \text{TransformationAmplitude[} & \\ & \text{<rhoini>}\to \text{\{<Qobs\#1>,<Qobs\#2>...\}}\\ & \text{<events>,<opts>}\\ & \text{]} \end{array}$$

By default, TransformationAmplitude calls NDSolve with the options InitialTimePoint→0 and FinalTimePoint→Automatic. The *t*=0 origin of the global time variable is therefore at the start of the sequence. This is only of importance in the case of time‐dependent generators.

Some examples of TransformationAmplitude are as follows.

#### 9.2.1 Single pulse

This example shows the effect of a finite‐duration (*π*/2)_*y*_ pulse on z‐magnetization:


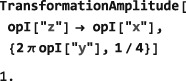


As expected, there are no contributions of *I*_*z*_ or *I*_*y*_ in the final density operator, and the overlap with *I*_*x*_ is maximal.

#### 9.2.2 Generation of antiphase terms

The example below shows that the density operator term *I*_1*x*_ is transformed into the antiphase term 2*I*_1*y*_ *I*_2*z*_ by evolution under a secular J‐coupling Hamiltonian. In the example below, the J‐coupling is 1 Hz, and the BackgroundGenerator argument is used to specify the J‐coupling Hamiltonian. The amplitude of the antiphase term reaches a maximum after half a second of evolution, while the amplitude of the in‐phase *I*_1*x*_ term goes to zero:


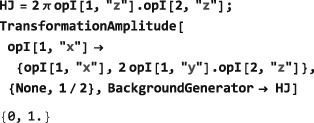


#### 9.2.3 Radio‐frequency field inhomogeneity

A common problem in magnetic resonance is *B*_1_ field inhomogeneity. This influences the nutation amplitude of a given pulse and leads to different flip angles in different spatial regions. The lines below illustrate how the RandomVariate and NormalDistribution routines of Mathematica may be used to generate 100 samples of a normal distribution with standard deviation 0.3 Hz, centered around 1 Hz.


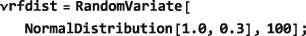


The effect of the amplitude variations on a simple (*π*/2)_*y*_ pulse may be simulated by using the EnsembleAverage option of TransformationAmplitude:


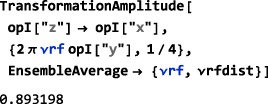


As expected, the efficiency of the transformation is reduced by the spread in rf field strengths.

### 9.3 **TransformationAmplitudeTable**

TransformationAmplitudeTable is an extension of TransformationAmplitude that allows iteration over a free parameter, by analogy with the Table routine of Mathematica. This may be used to assess the dependence of pulse sequence performance on a pulse sequence or interaction parameter.

The syntax is as follows:

$$\begin{array}{ccc} & \text{TransformationAmplitudeTable[} & \\ & \text{<rhoini>}\to \text{<Qobs>,<events>,}\\ & \text{\{<i>,<imin>,<imax>,<di>\},}\\ & \text{<opts>}\\ & \text{]} \end{array}$$

The order of the input arguments is quite strict in this case. The <events> and <opts> arguments may be functions of the iterator <i>. The iterator <i> takes a set of values starting with <imin> and incrementing by <di> up to a value <imax>. The output of TransformationAmplitudeTable is a list of TransformationAmplitude values, ordered according to the incrementation.

TransformationAmplitudeTable takes optional arguments for symbols such as EnsembleAverage, NormalizationFactor, and BackgroundGenerator. These arguments may also be a function of the iterator <i>.

A special optional argument unique to TransformationAmplitudeTable is TableCoordinates→<tabcoords>. The option value for <tabcoords> may depend on the iterator <i>. The resulting output is in the form of an xy‐list, where the y‐coordinates are given by the TransformationAmplitude values and the x‐coordinates correspond to the TableCoordinates expression. The resulting output is compatible with many plotting functions of Mathematica, such as ListPlot. The example below considers resonance offset effects on a *π*/2 pulse, applied to initial z‐magnetization. The residual z‐magnetization changes as the resonance offset mismatch changes from ‐1 to +1 kHz.

In this case, the option TableCoordinates→Ω/(2π) generates x‐coordinates, which are in units of Hz, even though the offset frequency Ω is in radians per second. In general, any expression containing the iterator may be used in the TableCoordinates option.


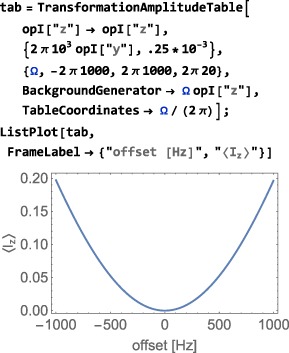


TransformationAmplitudeTable may also be used to determine the dependence of any number of target operators on a variable, at the same time. The required syntax is as follows:

$$\begin{array}{ccc} & \text{TransformationAmplitudeTable[} & \\ & \text{<rhoini>}\to \text{\{<Qobs\#1>,<Qobs\#2>...\}}\\ & \text{<events>,}\\ & \text{\{<i>,<imin>,<imax>,<di>\},}\\ & \text{<opts>}\\ & \text{]} \end{array}$$

### 9.4 **Trajectory**

Observing the time evolution of a given set of observables is often essential for understanding the dynamics of the system. SpinDynamica's Trajectory function may be used to generate such time trajectories.

The syntax of Trajectory is as follows:

$$\begin{array}{ccc} & \text{Trajectory[} & \\ & \text{<rhoini>}\to \text{<Qobs>,<events>,<opts>}\\ & \text{]} \end{array}$$

The output of Trajectory is not a number or a set of numbers, but a TrajectoryFunction. This function may be evaluated at any time point during the simulated event sequence.

As an illustration, consider the effect of a finite‐duration (*π*/2)_*y*_ pulse on initial z‐magnetization. Trajectory generates the following output:


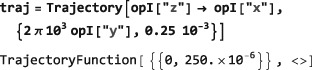


The output TrajectoryFunction is assigned to the symbol traj.

The TrajectoryFunction is a continuous function over a given time interval, indicated by the first argument after the square brackets. For this particular example, the TrajectoryFunction is defined over the interval *t*∈[0,0.25] ms. The TrajectoryFunction may be evaluated at any time point that is an element of the time interval.


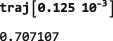


TrajectoryFunction objects may be treated in the same way as any other Mathematica function. For example, they may be plotted using Plot:


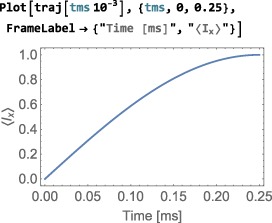


Some more examples of Trajectory are as follows.

#### 9.4.1 Composite pulse

When applied to a sequence of events, Trajectory generates a piecewise function, which handles the discontinuities at event junctions. For example, consider the following composite pulse(53):


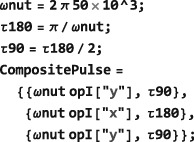


The overall rotation of the composite pulse corresponds to the inversion of z‐magnetization. The trajectories for the three magnetization components are calculated as follows:


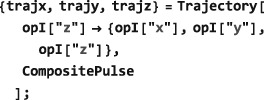


The trajectories of the three components may be plotted simultaneously:


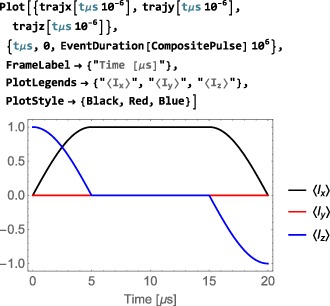


In this example, the function EventDuration has been used to extract the total duration of the composite pulse, and the time axis converted to units of *μ* *s*, for convenience. The composite pulse leads to the inversion of z‐magnetization, as expected.

#### 9.4.2 Chirp pulse

In many cases, it is desirable to perform broad‐banded and uniform inversion of the magnetization. The performance of inversion pulses may be improved by varying the radiofrequency field amplitude, the phase, or the rf frequency, as continuous functions of time.(54) The example below illustrates a Gaussian pulse with a maximum nutation frequency of 100 Hz, a constant phase, and a frequency swept according to a hyperbolic tangent function. Such a pulse is conveniently defined by using the ShapedPulse function (Section 8.3.3). The response of the x‐, y‐, and z‐magnetization components are simulated in the following manner.


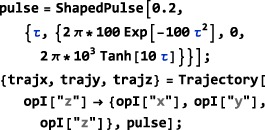


The magnetization trajectories take the following form:


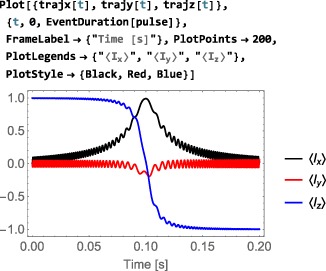


The trajectories reflect the fact that the radiofrequency field is initially far from resonance but sweeps through resonance at ≈100 ms, leading to fast inversion of the z‐magnetization.

The ParametricPlot3D function of Mathematica, and the graphics object SphereAndAxes of SpinDynamica, may be used to plot the trajectory of the tip of the magnetization vector in 3D space:


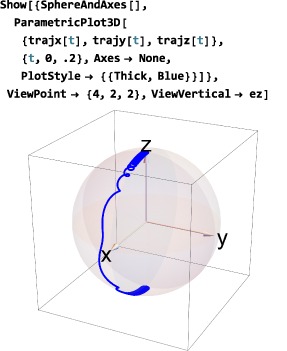


Most front ends of Mathematica allow interactive 3D rotation of such graphical objects on the screen.

#### 9.4.3 Inversion recovery

*T*_1_ relaxation times are often estimated by performing an inversion‐recovery experiment. Since this experiment involves the recovery of the density operator to thermal equilibrium, simulation of this experiment requires a rigorous treatment of the spin density operator, and use of a thermalized relaxation superoperator (see Sections 7.1 and 8.1.3).

For completeness, the implementation shown below includes a coherent Hamiltonian (here in the form of a simple resonance offset term), in addition to the relaxation terms. The calculation is prepared as follows: (a) A laboratory frame Hamiltonian Hlab is set up, describing the interaction of protons with a magnetic field of 11.4 Tesla along the z‐axis; (b) a rotating‐frame offset Hamiltonian HΩ is defined; (c) a phenomenological relaxation superoperator Γ is defined, corresponding to relaxation time constants *T*_1_=*T*_2_=1; (d) the relaxation superoperator is thermalized, using the defined laboratory frame Hamiltonian and a sample temperature of 300 Kelvin. This leads to the thermalized relaxation superoperator ΓΘ; (e) the thermal equilibrium density operator *ρ*Θ is defined under the same conditions; (f) the thermal equilibrium magnetization Meq is defined, and (g) the relaxation superoperator and offset Hamiltonian are combined to give the Liouvillian L:


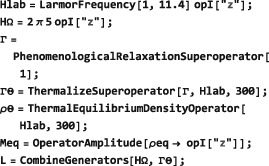


This sets the stage for simulation of an inversion‐recovery experiment, including a resonance offset. The event sequence for a single time point of the inversion‐recovery experiment, with interpulse delay of 0.25 s, is defined as follows:


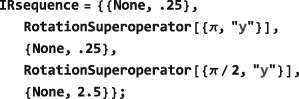


An initial delay of 0.25 s before the first pulse is included for the sake of clarity.

The simulated trajectories of the x‐ and z‐components of the magnetization are calculated and plotted as follows:


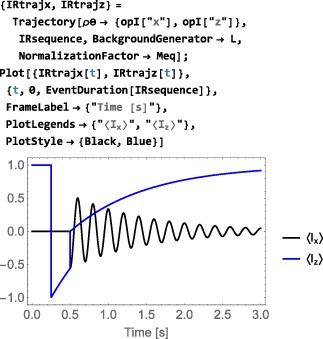


The trajectories are normalized by using the NormalizationFactor→Meq option, so that thermal equilibrium corresponds to unit z‐magnetization.

The plot shows the inversion and recovery of the z‐magnetization after the *π* pulse, the destruction of the z‐magnetization by the *π*/2 pulse, and the oscillatory decay of the transverse x‐magnetization induced by the *π*/2 pulse.

Other phenomena involving relaxation towards thermal equilibrium combined with coherent Hamiltonians may be simulated in a similar fashion. Examples include steady‐state relaxation phenomena(45) and the nuclear Overhauser effect.(55)

#### 9.4.4 Total correlation spectroscopy

The code in this example assumes prior execution of the command SetSpinSystem[3].

Total Correlation Spectroscopy (TOCSY) methods are often used to disentangle isolated groups of spins when similarities in chemical shifts would lead to spectral overlap and hence ambiguous resonance assignments.(2) Consider a three‐spin‐1/2 system arranged in a strongly coupled chain, defined by the following Hamiltonian:


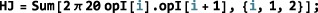


The coupling strength between each pair of spins equals 20 Hz. For such a system the following commutation relations hold:

(38) $$\begin{array}{ccc} & [H_{J},I_{\mu }]=0 & \\ & [H_{J},I_{j\mu }]\neq 0 \end{array}$$

where $I_{\mu }=\sum_{j}I_{j\mu }$ and *μ* specify a spatial direction. This implies that the total magnetization along any spatial axis is a constant of motion, while the magnetizations of individual spins are subjected to evolution.

The following calculation simulates the trajectories of the three z‐magnetization components and the total z‐magnetization, starting from a state in which only the first spin possesses z‐magnetization:


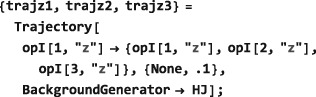


The trajectories are plotted below:


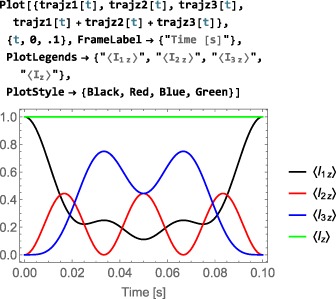


As can be seen, the magnetization of the three spins are exchanged in an oscillatory fashion, although the sum of all three components is a constant.

### 9.5 **Signal1D**

Signal1D is a large and versatile SpinDynamica routine for the generation of one‐dimensional time‐domain NMR signals (i.e., free‐induction decays, or FIDs). The routine incorporates a variety of methods for simulating the NMR signal, for specifying the time sampling of the signal, for specifying the initial density operator and the preparation sequence before data acquisition starts, and for specifying the generators, which act during the signal evolution and during the preparation sequence.

The basic syntax of Signal1D is given by:

Signal1D[<timing>,<acq>,<opts>]

The <timing> argument specifies how the FID is sampled; the <acq> argument specifies what goes on during the acquisition of the FID, and the <opts> argument contains the settings for numerous parameters, including specifications for what happens before the signal is acquired.

#### 9.5.1 The **<timing>** argument

The <timing> argument specifies the timing of the signal sampling. Several syntactical forms are available.

The most basic form of the <timing> argument has the form:

<timing> = {<tmin>,<tmax>,<dt>}

This specifies the initial time point <tmin> for the start of sampling, and the final time point <tmax> for the end of sampling. Both <tmin> and <tmax> are global time values (see Section 8.3.2). The parameter <dt> specifies the time increment between each sampling point, often known as the *dwell time*. If the initial time point <tmin> is omitted, a value of 0 is assumed.

In many cases, a more convenient syntax for the <timing> argument has the form

<timing> = {<tmin>,{<SW>,<NPoints>}}

Double curly brackets are necessary and should not be omitted. The <SW> argument sets the spectral width of the resulting spectrum (in angular frequency units, i.e., rad s^−1^). The number of sampling points, NPoints, may either be an integer or a string of the following type: “1k”, “2k”, “4k”, etc. If the <tmin> argument is omitted, a value of 0 is assumed. The Signal1D routine converts this syntax into the {<tmin>,<tmax>,<dt>} form internally before proceeding with the calculation.

For example, consider these two sampling specifications:


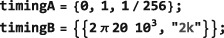


The first specification indicates that the sampling proceeds for 1 s, starting at *t*=0, with a dwell time of 1/256 s. The second specification indicates that sampling starts at *t*=0, with a sampling bandwidth of 20 kHz, using 2,048 sampling points.

#### 9.5.2 The **<acq>** argument

The <acq> argument specifies what happens during signal acquisition. Several forms are available:

- *No argument.* If <acq> is omitted, the null generator None is assumed.
- *Generator argument.* The <acq> argument may specify any generator of the type described in Section 8.1. This generator is assumed to act throughout the signal acquisition interval, in combination with the value of BackgroundGenerator as specified in the <opts> arguments (see Section 9.5.3).
  For example, the <acq> argument could be used to specify the spin Hamiltonian acting during the evolution of the FID.
  The generator specified in the <acq> argument may be time‐dependent. If periodic time‐dependence is indicated by using the PeriodicFunction symbol, special computational routines for periodic generators are deployed automatically by Signal1D (see Section 9.5.4).
- *Events argument.* The <acq> argument may specify a single event, or sequence of events (Section 8.3). The evolution of the density operator during the sampling of the FID takes place while these events are proceeding, possibly in combination with BackgroundGenerator (Section 9.5.3).
  If the specified events do not extend over the complete acquisition interval, Signal1D automatically pads the end of the event list with a delay event of the form {None,<duration>}.
- *Repeated events*. The special syntax Repeat[<events>] indicates that the event sequence <events> is repeated as many times as needed to fill the acquisition interval. This syntax usually triggers the use of special routines for signals evolving under periodic generators (see Section 9.5.4). This syntax is useful for the simulation of signal detection under periodic pulse sequences.(56)

#### 9.5.3 The optional arguments

The <opts> argument of Signal1D is a series of replacement rules specifying the values of optional parameters, that is <par#1>→<val#1>, <par#2>→<val#2>. As usual, these replacement rules may be given in any order.

Replacement rules may be given for the following parameters:

- InitialDensityOperator and Preparation.
Signal1D takes an initial density operator, specified by the InitialDensityOperator argument, and propagates it in time under the sequence of events specified by the Propagation argument. This density operator is then used as the starting point for evolution during the signal acquisition interval. The density operator at the start of signal acquisition therefore depends on the option values for both InitialDensityOperator and Preparation.
  The InitialDensityOperator argument may be any valid operator. If no value is specified, the default InitialDensityOperator→opI[“z”] is used, that is, z‐polarization for all spins in the current system.
  The Preparation argument may be any valid event or sequence of events (see Section 8.3). If no value is specified, the default Preparation→RotationSuperoperator[{ *π*/2,“x”}] is used, that is, an ideal *π*/2 pulse about the x‐axis for all spins in the system.
  The default situation is therefore that the initial density operator corresponds to *I*_*z*_ for all spins, which is rotated by *π*/2 about the x‐axis before data acquisition starts. By default, the density operator at the start of signal acquisition is therefore −*I*_*y*_ for all spins.
  In the case of time‐dependent generators (see Section 8.1.2), one should note that the rules FinalTimePoint→<tmin> and InitialTimePoint→Automatic are passed to the NPropagate routine when calculating the propagation of the initial density operator through the Preparation sequence. This implies that the global time variable takes the value *t*=<tmin> at the end of the preparation sequence.
- BackgroundGenerator
  The BackgroundGenerator argument of Signal1D specifies the background generator acting during the Preparation sequence, and also during the signal acquisition.
  If desired, different background generators for the preparation sequence and for the signal acquisition may be specified by using the syntax:
  $$\begin{array}{ccc} & \text{BackgroundGenerator}\to & \\ & \text{\{<prepbackg>,<acqbackg>\}} \end{array}$$
  The value of BackgroundGenerator value may be a time‐dependent Hamiltonian or Liouvillian. The use of PeriodicFunction as a value for BackgroundGenerator usually triggers the use of special routines for signals evolving under periodic generators (see Section 9.5.4). This syntax is often used for the simulation of NMR signals in magic‐angle‐spinning NMR.
- Observable
  The Observable argument of Signal1D may be any spin operator.
  In addition, the special forms Observable→<lab> and Observable→{<lab#1>,<lab#2>…} are available, where the label arguments refer to the members of the SpinSystem (see Section 4.1). This syntax indicates the selective observation of one or more types of spins {*I*_*j*_,*I*_*k*_…}. The observable operator is defined as follows:
  (39) $$Q_{\text{obs}}=-\frac{1}{2}i(I_{j}^{-}+I_{k}^{-}+\text{⋯})$$
  This form of observable operator leads to the acquisition of a complex NMR signal, corresponding to conventional quadrature detection.(2)
  For example, after setting up a three‐spin‐1/2 system by the instruction SetSpinSystem[3], execution of Signal1D with the option Observable→{1,2} indicates that only the first two spins in the system are detected.
  If the Observable argument is omitted, the default Observable→All is used. This causes all spins in the current system to be observable, with the observable operator defined by Equation 39.
  Specifying a subset of spins as being observable is usually essential when simulating heteronuclear NMR spectra.
- EnsembleAverage
  The EnsembleAverage argument may be used to trigger the linear combination of multiple Signal1D simulations, with one or more symbols taking different values, as specified in Section 9.1.2. An example is shown below (Section 9.5.8).
- CarouselAverage
  The CarouselAverage option may be used in conjunction with periodic calculation algorithms (see Section 9.5.4).
- LineBroadening
  The LineBroadening argument may be used to specify an artificial exponential decay applied to the simulated FID. The value of LineBroadening specifies the full‐width‐at‐half‐height of the Lorentzian peak obtained by a Fourier transform of the decaying signal, in angular frequency units (rad s^−1^).
  For example, the option
  LineBroadening→2π×10
  specifies a full‐width‐at‐half‐height of 10 Hz for the frequency‐domain spectrum, in the absence of other damping mechanisms.
  In general, an option value LineBroadening→*λ* causes the simulated FID to be multiplied by the time‐domain function $\exp\{-\lambda (t-t_{0})/2\}$, where *t*_0_ is the value of the <tmin> argument of Signal1D.
  The option LineBroadening→None suppresses the artificial line broadening.
  The default option is LineBroadening→Automatic, which multiplies the FID by an exponential decay function starting from 1 at the start of the FID, and decaying to 0.01 at its end. This is usually sufficient to minimize spectral truncation artifacts with a minimum of fuss.

#### 9.5.4 Calculation methods

Signal1D deploys a variety of calculation methods for the NMR signal. The appropriate method used is selected automatically based on the forms of the <acq> argument and the BackgroundGenerator option, although the user may override this (see the SpinDynamica documentation for details).

The available calculation methods are as follows:

- *Diagonalization*
Signal1D selects a diagonalization algorithm in the case that the relevant generator is time‐independent over the entire data acquisition interval.(39) The diagonalization is performed in Hilbert space for time‐independent Hamiltonians, and in Liouville space for time‐independent Liouvillian generators.
- The *COMPUTE* algorithm.
Signal1D deploys the COMPUTE (Computation over One Modulation Period Using Time Evolution) algorithm for calculating the evolution under periodic time‐dependent generators. A Hilbert space algorithm is used for periodic Hamiltonians, (40, 57)while a Liouville space algorithm is used for periodic Liouvillians.(41) In addition, the option CarouselAverage→True may be used in conjunction with the COMPUTE algorithm to perform a rapid average over the phase of the periodic generator.(42, 58, 59)
- *Direct method.*
  If the other methods are not suitable, Signal1D uses Trajectory to calculate the evolution of the observable during the signal acquisition interval and then samples the computed trajectory at the appropriate time points. This method is general but is usually slower than the other algorithms.

#### 9.5.5 The **Signal** output

The code in this section assumes prior execution of the command SetSpinSystem[2].

Signal1D generates a Signal object. This object contains the information needed to generate a set of {<time>,<amplitude>} data points, for further processing and plotting.

For example, after SetSpinSystem[2], the following instruction sets up the Hamiltonian for a two‐spin system with resonance offsets 200 and 300 Hz, and a J‐coupling of 50 Hz, and assigns the result to the symbol H:


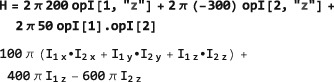


Simulation of the FID, with a sampling bandwidth of 1 kHz and 2,048 sampling points, using default values of the Preparation and InitialDensityOperator parameters, is performed as follows:


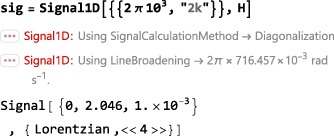


The Signal1D routine reports the calculation algorithm that has been used (in this case, diagonalization), and that a Lorentzian line broadening of 0.71 Hz has automatically been applied. The output of the Signal1D routine is a Signal object, which is assigned to the new symbol sig. The first part of the formatted output indicates that the Signal object extends from time points 0 to 2.046 s, with a sampling interval of 1 ms. The second part indicates the presence of four Lorentzian peaks, as expected for a AB spectrum.

If desired, the Signal object may be converted to a list of {<time>,<amplitude>} pairs by applying Expand. The code below applies Expand to the signal object and displays the first 5 points:


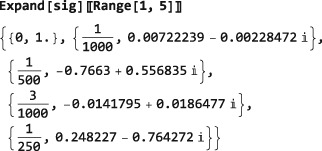


This shows the sampling time points and complex signal amplitudes explicitly.

#### 9.5.6 Plotting the FID

The real part of the FID may be plotted by using the Mathematica routines ListPlot and Re:


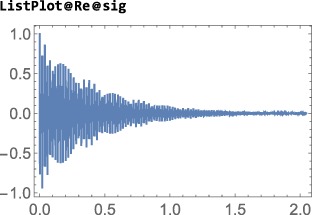
 which shows a conventional spectrometer‐like FID display. The imaginary part of the FID may be plotted by using Im instead of Re.

#### 9.5.7 Fourier transformation

A spectral display of the signal requires Fourier transformation. Mathematica contains the native routine Fourier, but this proves to be inconvenient to use directly. The SpinDynamica routine FT may be applied to a Signal object and generates a result containing appropriate frequency coordinates, which may be plotted directly. The following code takes the Fourier transformation of the signal and plots the real part:


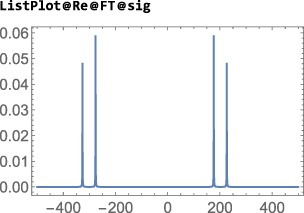


This shows the expected AB spectral pattern.

#### 9.5.8 An example of **EnsembleAverage**

The EnsembleAverage option may be used to average signals over any desired parameter, or set of parameters. In the example below, an ensemble of spins‐5/2 is set up, and a quadrupole coupling Hamiltonian defined, which depends on the quadrupolar splitting parameter *ω*_*Q*_:


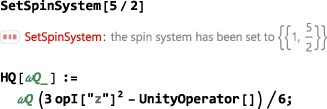


As in the example given in Section 9.2.3, the lines below use the RandomVariate and NormalDistribution routines of Mathematica to take 1,000 samples of a normal distribution with standard deviation 30 Hz, centered around 1,000 Hz. The EnsembleAverage instruction causes SpinDynamica to repeat the Signal1D calculation with these values for the quadrupolar splitting, combine the results, Fourier transform and plot:


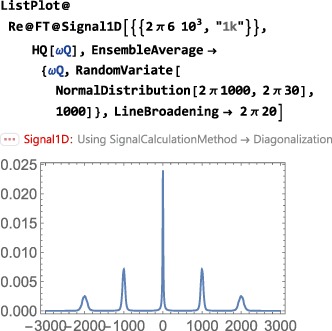


The resulting spectrum shows increased broadening of the outer satellite peaks, and a narrow central transition.

This brief example shows that SpinDynamica can implement relatively sophisticated physical models with a minimum of user coding.

## 10 ADDITIONAL FUNCTIONALITY

SpinDynamica provides a substantial amount of additional functionality, which may only be described briefly here. More information is available in the SpinDynamica documentation.

### 10.1 Spin interactions

Routines such as SpinQuantumNumber, NaturalAbundance, GyromagneticRatio, LarmorFrequency, DirectDipolarCoupling, and PhysicalConstants facilitate the calculation of common spin interaction parameters. SpinDynamica contains tables of gyromagnetic ratios for all magnetic nuclides, which may be indicated either by their mass number, or as a string of the form “23Na”. For example, the Larmor frequency of ^23^Na in a field of 11.4 Tesla, in units of rad s^−1^, is obtained as follows:


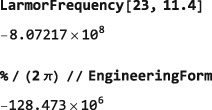


This shows that ^23^Na nuclei precess in the negative sense at 128.5 MHz in a field of 11.4 Tesla. The sign of the Larmor frequency has a physical meaning.(2, 60)

### 10.2 3D geometry

Routines such as SubtendedAngle, BondAngle and Distance facilitate the calculation of geometrical parameters from molecular coordinates. The routine Axes3D facilitates the construction of a three‐dimensional right‐handed axis system, given one or two guide vectors (one vector determines the direction of the z‐axis in the new axis system, while a second, optional vector may be used to specify the plane in which the x‐axis of the new axis system lies).

### 10.3 Euler angles

The routine AxesToEuler derives the set of Euler angles, which define the relative orientation of two axis systems. The routines AxesToEuler and Axes3D are often used in combination to derive the Euler angle sets needed for spin dynamical calculations, based on sets of nuclear coordinates and models of interaction tensor orientations. The routine RotateEuler may be used to rotate objects in three‐dimensional space, using Euler angles to specify the 3D rotation. The Euler angle routines used by SpinDynamica conform to the common conventions of the NMR community.(39)

### 10.4 Wigner matrices

The Wigner matrices are irreducible representations of the full rotation group *S* *O*(3), denoted *D* ^*J*^(Ω), where *J* is the angular momentum quantum number (rank) and the orientation definition Ω={*α*,*β*,*γ*} is an Euler angle triplet. Wigner matrices are used heavily in the formulation of spin Hamiltonians.(30, 31, 32, 33)

The elements of a Wigner matrix are written

(40) $$D_{mm^{\prime }}^{J}(\alpha ,\beta ,\gamma )=\exp\{-im\alpha \}d_{mm^{\prime }}^{J}(\beta )\exp\{-im^{\prime }\gamma \}$$

where the reduced Wigner matrix is denoted *d* ^*J*^(*β*). The Wigner matrices of rank *J* are (2*J*+1)×(2*J*+1) square matrices.(61)

The SpinDynamica routines WignerD and Wignerd generate Wigner matrices and reduced Wigner matrices, respectively. Wigner matrices are frequently encountered in spin dynamical theory in the context of frame transformations. The syntax WignerD[2][{a,b,g}] provides a Wigner matrix of rank 2, while the syntax WignerD[2,{1,‐1}][{a,b,g}] provides a single element of the same Wigner matrix with *m*=1 and $m^{\prime }=-1$.

WignerD implements a concise syntax for consecutive frame transformations. For example, the instruction

WignerD[2,{1,‐1}][{{a1,b1,g1},{a2,b2,g2}}]

generates the $\left\{m,m^{\prime }\}=\{1,-1\right\}$ element of the product matrix *D* ^*J*^(*α*_1_,*β*_1_,*γ*_1_)*D* ^*J*^(*α*_2_,*β*_2_,*γ*_2_). This may be extended to any number of consecutive transformations.

Awkwardly, a native Mathematica implementation of WignerD was introduced in Mathematica version 10, but with a different Euler angle convention. Fortunately, the syntax used by SpinDynamica does not conflict with the native Mathematica syntax: An explanatory warning is generated by SpinDynamica the first time WignerD is used.

### 10.5 Clebsch‐Gordan coefficients

Mathematica includes the native routine ClebschGordan, which generates the angular momentum coupling parameters. However, the conventions used by Mathematica are again in conflict with common conventions used in NMR theory. The SpinDynamica routine CG implements the standard NMR conventions.

### 10.6 Matrix routines

SpinDynamica includes a set of matrix/vector utility routines such as DirectProduct, Adjoint, and BlockDiagonalMatrix. The routine NDiagonalize is heavily used by the internal code of SpinDynamica and converts a matrix into a diagonalized form suitable for processing by many other routines.

### 10.7 Graphical objects

SpinDynamica contains code for some useful graphical objects, such as Axes3D and SphereAndAxes. These are useful for depicting trajectories in 3D space.

## 11 CONCLUSIONS

SpinDynamica is a work‐in‐progress. At the time of writing, there are some prominent gaps in the high‐level functionality. At the moment, there is no general top‐level routine for multidimensional spectroscopy, or for handling chemical exchange—although custom code exploiting SpinDynamica routines has been written for some special cases.(12) In addition, a great deal of work remains to be done in improving the computational efficiency of the low‐level routines and in exploiting the powerful pattern‐matching capabilities of Mathematica to avoid duplicated calculations.

SpinDynamica currently requires the user to program Hamiltonians and relaxation superoperators explicitly; a set of high‐level routines for rapidly constructing such interaction terms for typical spin systems would be welcomed by many users. A translator from “pulse‐sequence syntax” to SpinDynamica events is in progress and will be fully implemented in the near future (see Section 8.3.3). The implementation of approximate algorithms such as operator basis restriction(62, 63) would allow SpinDynamica to handle larger spin systems than is currently possible. A general system for interpolating between the discrete calculations in EnsembleAverage could greatly accelerate the calculations of NMR powder averages(9, 64) and/or spatial variables, as in magnetic resonance imaging or spatially encoded NMR experiments.(65, 66) Although SpinDynamica may handle very complex relaxation effects, such as anisotropic molecular tumbling, internal molecular rotation, and cross‐correlation, an accelerated procedure for setting up relaxation superoperators(67) would be very useful. Coding for chemical exchange and other phenomena requiring extended Liouville bases is in progress but not yet implemented at the time of writing. Another missing feature is a set of general routines for importing spectrometer data into SpinDynamica (some users have written such code, but not yet made it available to the community, as far as we know).

Despite these clear limitations, most of the existing SpinDynamica routines are highly general and robust and provide a general platform upon which users may build their own modules and solve specific problems in magnetic resonance theory, analysis, and simulation. The authors hope that this article will stimulate users to construct add‐on packages, for distribution through the SpinDynamica website and forum (www.spindynamics.org). Sufficiently general and robust packages will be incorporated (with acknowledgments!) into future SpinDynamica releases.

## Data Accessibility

## APPENDIX A USE OF MATHEMATICA

### A.1

#### A.1.1

*Notebooks, Cells, and Kernels*

Executable and interactive Mathematica files are called *notebooks* (extension .nb). Notebooks are organized into *cells*. The following example shows two Mathematica cells.

The content of a given cell is enclosed by the gray bracket to the right. Moving between cells can be done by either using the <up> and <down> keys or mouse navigation.

Inexperienced users do not find it immediately obvious how to execute a Mathematica cell. This is conveniently done by selecting the cell and keying <shift> and <enter> at the same time.

Executing any cell starts the Mathematica *kernel*, which is the computational engine. It is important to understand that the results of all computations, symbols assignments, etc. are retained by the kernel. This applies even if new Mathematica windows are opened in the user interface (called the *Front End*). In normal circumstances, all front end windows interact with the same kernel.

The kernel may be quit and all symbols cleared through the Front End menu selection Evaluation > Quit Kernel > Kernel.

*Basic Syntax*

Symbols: Mathematica is based on the manipulation of *symbols*. An example of symbol manipulation is as follows: In most front ends, symbols are displayed in blue, until they have assigned values, when they turn black, as shown below: The assigned symbol may be used for further calculations: The use of = assigns a value to a symbol immediately. A slightly different effect is achieved by a *delayed assignment*, achieved by using a := construction. In this case, the assignment is made when the symbol is used, rather than immediately. The following examples illustrate the subtle difference: The ; characters are used to suppress to the output of certain lines.
Greek: A greek letter may be entered by typing <esc><first letter><esc>. For example, *α* is entered by typing <esc>a<esc>.
Rule: transformation rules may be declared using Rule, as follows: Rule[a,b] which is interpreted as a goes to b. A Rule character may also be defined using a→b The → is entered by typing <esc> ‐> <esc> (most Mathematica front ends automatically replace ‐> by →).
ReplaceAll: A Rule is applied to a set of symbols by using the instruction ReplaceAll, which takes the following syntax: ReplaceAll[<input>,a→b] An example is as follows: This instructs Mathematica to replace all instances of z by Sqrt[x^2+y^2]. ReplaceAll may also be implemented by a construction using the characters /., as follows: Mathematica's array structures are called List objects and are generated by a sequence of symbols separated by commas and enclosed by curly brackets, for example {a,b,c}.
Part: The extraction of elements from a list is performed by using Mathematica's Part function. The first argument is the list in question, while the second argument indicates the position of the desired element. The same operation may be conducted as follows: The double brackets may be entered by typing <esc>[[<esc> and <esc>]]<esc> respectively. The same result is achieved by omitting the <esc> characters, but the output is less readable.
Options: Many Mathematica functions are able to handle optional input arguments. The default options for a given symbol may be inspected by executing: Options[<function>] The default options may be superseded by making use of the Rule functionality. They follow the general pattern: <name>→<value> where <name> is the name of an optional symbol and <value> is its value. Such option rules are usually given as the last arguments of a function and may be written in any order, that is, $$\begin{array}{ccc} & \text{<function>[} & \\ & \;\text{<arg>,<name\#1>}\to \text{<value\#1>,}\\ & \;\text{<name\#2>}\to \text{<value\#2>,...}\\ & \;\text{]} \end{array}$$ Default option values may be set for any symbol by executing $$\begin{array}{ccc} & \;\text{SetOptions[<function>, <name\#1>}\to & \\ & \text{<value\#1>]} \end{array}$$
Question Mark: Evaluating a symbol prefixed by ? generates information about the symbol, as shown below for List: For native Mathematica symbols, clicking the » symbol accesses more information and examples. These may also be accessed directly through front‐end menu selections.

Needless to say, Mathematica contains an enormous number of functions and symbols. The small summary above is purely intended to help novice users of Mathematica get off the ground.
